# Sex-specific lipid dysregulation in the *Abca7* knockout mouse brain

**DOI:** 10.1093/braincomms/fcac120

**Published:** 2022-05-11

**Authors:** YuHong Fu, Ying He, Katherine Phan, Russell Pickford, Young-Bum Kim, Nicolas Dzamko, Glenda M. Halliday, Woojin Scott Kim

**Affiliations:** 1 Brain and Mind Centre, The University of Sydney, Sydney, NSW 2050, Australia; 2 Faculty of Medicine and Health, School of Medical Sciences, The University of Sydney, Sydney, NSW, Australia; 3 Bioanalytical Mass Spectrometry Facility, University of New South Wales, Sydney, NSW, Australia; 4 Division of Endocrinology, Diabetes, and Metabolism, Beth Israel Deaconess Medical Center and Harvard Medical School, Boston, MA, USA; 5 School of Medical Sciences, University of New South Wales & Neuroscience Research Australia, Sydney, NSW, Australia

**Keywords:** Alzheimer’s disease, ABCA7, lipid dysregulation, lipidomics, cholesterol

## Abstract

Alzheimer’s disease is a devastating neurodegenerative disease that affects more women than men. The pathomechanism underlying the sex disparity, especially in the brain, is unclear. *ABCA7* is one of the strongest susceptibility genes for Alzheimer’s disease. It mediates the transport of lipids across membranes and is associated with pathways related to amyloid-β neuropathology. However, the role of ABCA7 in the regulation of brain lipids is largely unknown. Sex-specific differences in the pathological link between brain lipid dysregulation and amyloid-β are also unknown. Here, we undertook quantitative discovery lipidomics of male and female *Abca7* knockout (*n* = 52) and wild type (*n* = 35) mouse brain using sophisticated liquid chromatography/mass spectrometry. We identified 61 lipid subclasses in the mouse brain and found sex-specific differences in lipids that were altered with *Abca7* deletion. The altered lipids belong to cellular pathways that control cell signalling, sterol metabolism, mitochondrial function and neuroprotection. We also investigated the relationship between lipids and amyloid-β levels in the *Abca7* knockout mice and found elevated free cholesterol only in female mice that was significantly correlated with amyloid-β42 levels. In male *Abca7* knockout mice, the neuroprotective ganglioside GD1a levels were elevated and inversely correlated with amyloid-β42 levels. Collectively, these results demonstrate that *Abca7* deletion leads to sex-specific lipid dysregulation in the brain, providing insight into the underlying sex disparity in the aetiology of Alzheimer’s disease.

## Teaser

Fu *et al*. report sex-specific differences in brain lipids that are trafficked through the Abca7 transporter, suggesting a potential basis for the sex-specific differences in the aetiology of Alzheimer’s disease.

## Graphical Abstract

**Graphical Abstract:**
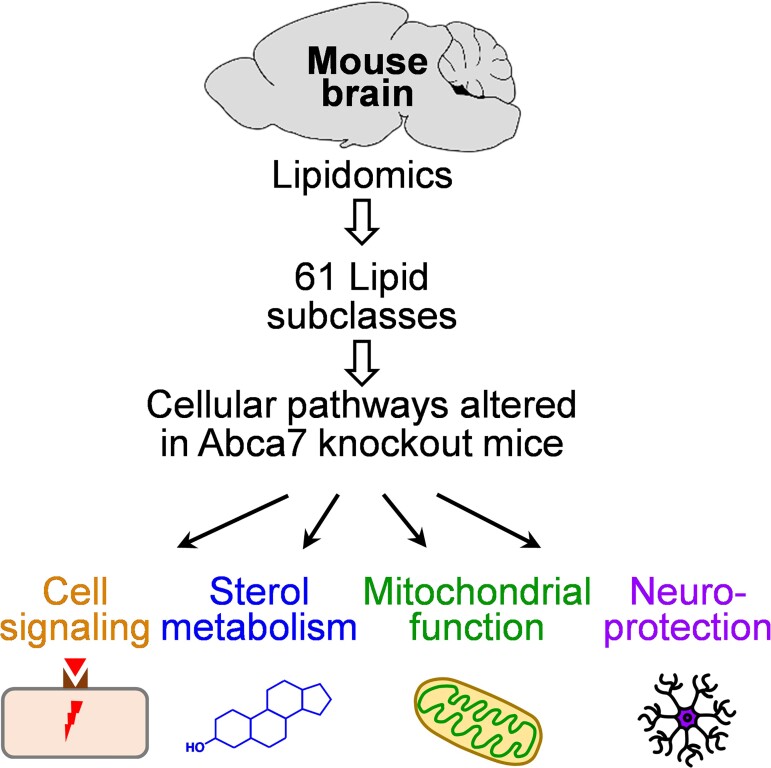

## Introduction

Alzheimer’s disease is an overwhelming neurodegenerative disease with the aetiology of the majority of cases is unknown. Alzheimer’s disease impacts on brain regions that control cognition and many physical functions. Approximately two-thirds of people with Alzheimer’s disease are women.^1^ This was originally thought to be due to women living longer than men. However, when the incidence of Alzheimer’s disease is adjusted for age, the risk is still higher in women than men.^2,3^ Indeed, the estimated lifetime risk for Alzheimer’s disease at age 45 is 19.5% in women and 10.3% in men, and at age 65 it is 21.1% in women and 11.6% in men.^4,5^ Transcriptional data also show that the disease burden of Alzheimer’s disease is greater in women, even after correcting for age and pathology.^6^ Women carrying one or more *APOE* ε4 alleles are more likely to develop Alzheimer’s disease than men.^7–9^ Consistent with a sex difference in *APOE* genotype, sex disparity is also evident in genetic variation in other lipid metabolism pathways [i.e. high-density lipoprotein (HDL)-mediated lipid transport, lipoprotein metabolism, and lipid digestion, mobilization and transport].^10^ Furthermore, the clinical symptoms of Alzheimer’s disease are more severe in women than men,^11,12^ although the pathomechanisms underlying this are poorly understood. This is despite knowledge of clear sex differences in metabolism and brain structure.

One of the strongest susceptibility genes for Alzheimer’s disease is the ATP-binding cassette subfamily A member 7 (*ABCA7*).^13–15^ ABCA7 specializes in transporting lipids across cellular membranes, including cholesterol and phospholipids.^16^ Mutations in members of the ABCA subfamily are known to cause disorders characterized by dyslipidemia and abnormal accumulation of lipids in cells.^17^ ABCA7 impacts on amyloid-β neuropathology via multiple pathways, including modulating phagocytic clearance of amyloid-β peptides,^18^ and regulating amyloid precursor protein (APP) processing to generate amyloid-β peptides.^19^ A study from the Alzheimer’s disease neuroimaging initiative showed that ABCA7 had the strongest association with amyloid-β deposition, even stronger than APOE, and that the association was significant in the early stages of the disease, suggesting ABCA7 is associated with rapid amyloid-β accumulation.^20^

Little is known about the association or impact of ABCA7 in relation to sex. In one genetic association study, the single-nucleotide polymorphism (SNP) rs3764650 in the *ABCA7* gene was significantly associated with cognitive decline only in females.^21^ In another study, 15 SNPs within 500 base pairs upstream or downstream of the *ABCA7* gene were linked to Alzheimer’s disease risk in a sex-specific manner with 10 SNPs protective only in females.^22^ These observations are further supported by animal studies showing that deletion of *Abca7* caused decreases in total serum and HDL cholesterol only in female mice.^23^ Moreover, deletion of *Abca7* caused decreases in performance in a spatial reference memory test only in female mice.^24^ Thus, it appears that loss of function of ABCA7 has a sex-specific impact on certain physiological and/or cognitive functions.

Despite the importance of lipid dysregulation in Alzheimer’s disease pathogenesis and the fact that ABCA7 is a lipid transporter and is strongly associated with Alzheimer’s disease, the role of ABCA7 in the regulation of brain lipids is largely unknown. Moreover, any sex-specific differences in the pathological link between brain lipid dysregulation and amyloid-β are also unknown. In this study, we undertook quantitative discovery lipidomics to identify lipids that are altered in the brains of *Abca7* knockout (KO) mice, both male and female mice side-by-side. Here, we show that *Abca7* deletion leads to sex-specific lipid dysregulation in the brain, providing insight into the pathomechanism underlying the sex differences in the aetiology of Alzheimer’s disease.

## Materials and methods

### Animals

*Abca7* KO (*Abca7*^–/–^) mice were generated by Kim *et al.*^23^ They were established on the C57BL/6J background and backcrossed 19 generations. *Abca7*^–/–^ mice are homozygous for the transgene and the genotype was confirmed by Southern hybridization and polymerase chain reaction (PCR).^23^ Age-matched (∼5 months) wild type (WT) C57BL/6J mice were used as non-transgenic controls. Mice were kept in polysulfone cages (2–4 mice/cage) under a 12:12 h light:dark schedule with food and water available *ad libitum*. Each cage provided the same environment and featured a polycarbonate igloo and nesting material. Research and animal care procedures were approved by the University of New South Wales Animal Care and Ethics Committee in accordance with the Australian Code of Practice for the Care and Use of Animals for Scientific Purposes.

### Brain tissue processing

Mice were fasted overnight and on the following morning they were injected (i.p.) with a lethal dose of pentobarbitone sodium (0.24 mg/g body weight) and perfused through the left ventricle with phosphate buffered saline (PBS). Brain tissues were collected, and snap frozen in liquid nitrogen, and kept at −80°C for RNA and protein analyses. For histological analysis, mice were perfused with PBS and then 4% paraformaldehyde. Brains were fixed in 4% paraformaldehyde overnight and then cryoprotected in 30% sucrose prior to sectioning.

### Histology

The fixed brain tissues were embedded in paraffin as described previously.^25^ The 5 μm sections were prepared and stained with haematoxylin and eosin (H&E). Five representative photos of each section were taken at 20× magnification with an Axioskop microscope (Zeiss Germany) using an AxioCAM Mrc camera. The images were stored in uncompressed 24-bit colour TIFF format and analyzed using Image J based Adiposoft software as described previously.^26^

### *In situ* hybridization

Briefly, paraffin-embedded slides were prepared from the 4% paraformaldehyde-fixed tissues. They were then hybridized with *Abca7* sense and antisense riboprobes that were generated using digoxigenin-labelled UTP. Hybridization was performed at 65°C for 6 h, and the slides were washed in 0.1 × saline sodium citrate at 75°C for 6 min. The hybridized probe was detected with biotinylated anti-digoxigenin antibody, streptavidin-alkaline phosphatase conjugate and the substrate nitro blue tetrazolium/5-bromo-4-chloro-3-indolyl phosphate. The slides were counterstained with nuclear Fast Red.

### Lipid extraction and liquid chromatography/mass spectrometry

Lipid extraction and liquid chromatography/mass spectrometry were carried out following our previously published methods.^27–29^ Relative abundance of lipids was obtained from peak areas normalized to internal standards and data were analyzed using LipidSearch software 4.1.16.

### Thin-layer chromatography

Firstly, lipid concentrations were determined using the Sulfo-Phospho-Vanillin method, as previously described.^30^ Briefly, 10 µl of lipid extracts were loaded into a microplate and the solvent removed by evaporation at 90°C. Concentrated sulphuric acid (100 µl) was added and incubated for 20 min at 90°C. The plate was rapidly cooled to room temperature and background absorbance measured at 540 nm. Fifty microliters of vanillin-phosphoric reagent [20% (w/v) vanillin in 17% (v/v) phosphoric acid] was added, the colour developed for 10 min and absorbance measured at 540 nm. Total lipid was calculated based on a standard curve generated using fish oil (Blackmores). The lipids were then separated using thin-layer chromatography (TLC). Briefly, volumes of extracted lipids equivalent to 7.5 µg were spotted onto TLC plates (Silca gel 60, Merck). The plates were developed in two stages; firstly, in chloroform/methanol/water (40:10:1) to 5 cm, then by n-hexane/diethyl ether/acetic acid (65:35:1) to the top of the plates. Lipids were visualized by misting the plate with 5% (w/v) CuSO_4_ in 15% (w/v) H_3_PO_4_ and heating for 10 min at 180°C. Band intensities were determined using BioRad Image Lab.

### Protein extraction

Tris-buffered saline (TBS) and sodium dodecyl sulfate (SDS)-soluble proteins were serially extracted from 100 mg of fresh-frozen brain tissues, as previously described.^31^ Briefly, tissues were homogenized in 10 volumes of TBS homogenization buffer (20 mM Tris, 150 mM NaCl, pH 7.4, 5 mM EDTA, 0.02% sodium azide) containing protease inhibitor cocktail (Roche) using Qiagen TissueLyser (3 × 30 s, 30 Hz cycles), followed by centrifugation at 100 000 *g* for 1 h at 4°C, with supernatant collected as TBS-soluble fraction. The pellet was resuspended in SDS solubilization buffer (TBS homogenization buffer containing 5% SDS) using 3×30 s, 30 Hz cycles with TissueLyser, and centrifuged at 100 000 *g* for 30 min at 25°C, with supernatant collected as SDS-soluble fraction. Total proteins were extracted from 50 mg of mouse brain tissues with 250 µl of ice-cold hypotonic buffer [250 mM sucrose, 10 mM HEPES (pH 7.4), 1 mM EDTA] containing protease inhibitor cocktail (Roche). Tissues were homogenized using Qiagen TissueLyser (30 Hz, 3 × 30 s cycles), centrifuged at 800 *g* for 10 min at 4°C to remove cell debris. The supernatants were transferred to new tubes and stored at −80°C. Protein concentration was measured using a bicinchoninic acid assay (Pierce BCA Protein Assay Kit) following the manufacturer’s instructions.

### Free cholesterol assay

Free cholesterol (FC) was measured using Cholesterol Quantitation Kit (MAK043, Sigma-Aldrich) following the manufacturer’s instructions.

### Western blotting and ELISA

Protein extracts (10 µg) were heated with sample buffer (3.2% SDS, 32% glycerol, 0.16% bromophenol blue, 100 mM Tris–HCl, pH 6.8, 8% 2-mercaptoethanol). They were then electrophoresed on Criterion Stain-free 4–20% SDS–PAGE gels (Bio-Rad) and transferred onto nitrocellulose membranes at 100 V for 30 min. The membranes were blocked with TBS containing 5% nonfat dry milk and probed overnight at 4°C with the following antibodies: Amyloid Precursor Protein (Sigma-Aldrich, A8717, 1:1000), Presenilin 1 (Santa Cruz, sc-365450, 1:1000) and β-actin (Abcam, ab6276, 1:10 000). They were then washed three times in TBS containing 0.1% Tween 20 and incubated with horseradish peroxidase-conjugated secondary antibodies for 2 h at room temperature. Signals were detected using enhanced chemiluminescence and Gel Doc System (Bio-Rad). The signal intensity was quantified using Image Lab (Bio-Rad) and NIH ImageJ software (v1.45s). ELISA of amyloid-β40 (KMB3481, Thermo Fisher Scientific Australia) and amyloid-β42 (KMB3441, Thermo Fisher Scientific Australia) were carried out following the manufacturer’s protocol.

### RNA extraction and quantitative PCR

RNA was isolated using TRIzol reagent (Invitrogen) following the manufacturer’s protocol. All procedures were carried out using RNase-free reagents and consumables. One microgram of RNA was reverse transcribed into cDNA using Moloney-murine leukaemia virus reverse transcriptase and random primers (Promega, Madison, WI, USA) in 20 μl reaction volume. Quantitative PCR (qPCR) assays were carried out using a CFX Connect Real-Time PCR System (BioRad, Australia) and the fluorescent dye SYBR Green (Bio-Rad), following the manufacturer’s protocol. Briefly, each reaction (20 μl) contained 1× mastermix, 5 pmoles of primers and 1 μl of cDNA template. Amplification was carried out with 40 cycles of 94°C for 15 s and 60°C for 1 min. Gene expression was normalized to the geometric mean of three housekeeper genes, GAPDH, β-actin and 18S. A no-template control was included for each PCR amplification assay. The level of expression for each gene was calculated using the comparative threshold cycle (Ct) value method using the formula 2^−ΔΔCt^ (where ΔΔCt = ΔCt sample − ΔCt reference).

### Statistical analysis

Statistical analyses were performed using SPSS Statistics software (IBM, Chicago, IL, USA). Multivariate analyses (general linear model) were used to determine differences in lipid and protein levels in *Abca7* KO and WT mice with *post hoc* statistical signiﬁcance set at *P* < 0.05. Pearson’s correlations were used to determine if changes in measurements were associated with each other with statistical signiﬁcance set at *P* < 0.05.

### Data availability

Lipidomics raw data were generated at the Bioanalytical Mass Spectrometry Facility, University of New South Wales. Derived data supporting the findings of this study are available from the corresponding author, upon reasonable request.

## Results

### Sex-specific differences in the impact of *Abca7* deletion on brain lipids

**Figure 1:**
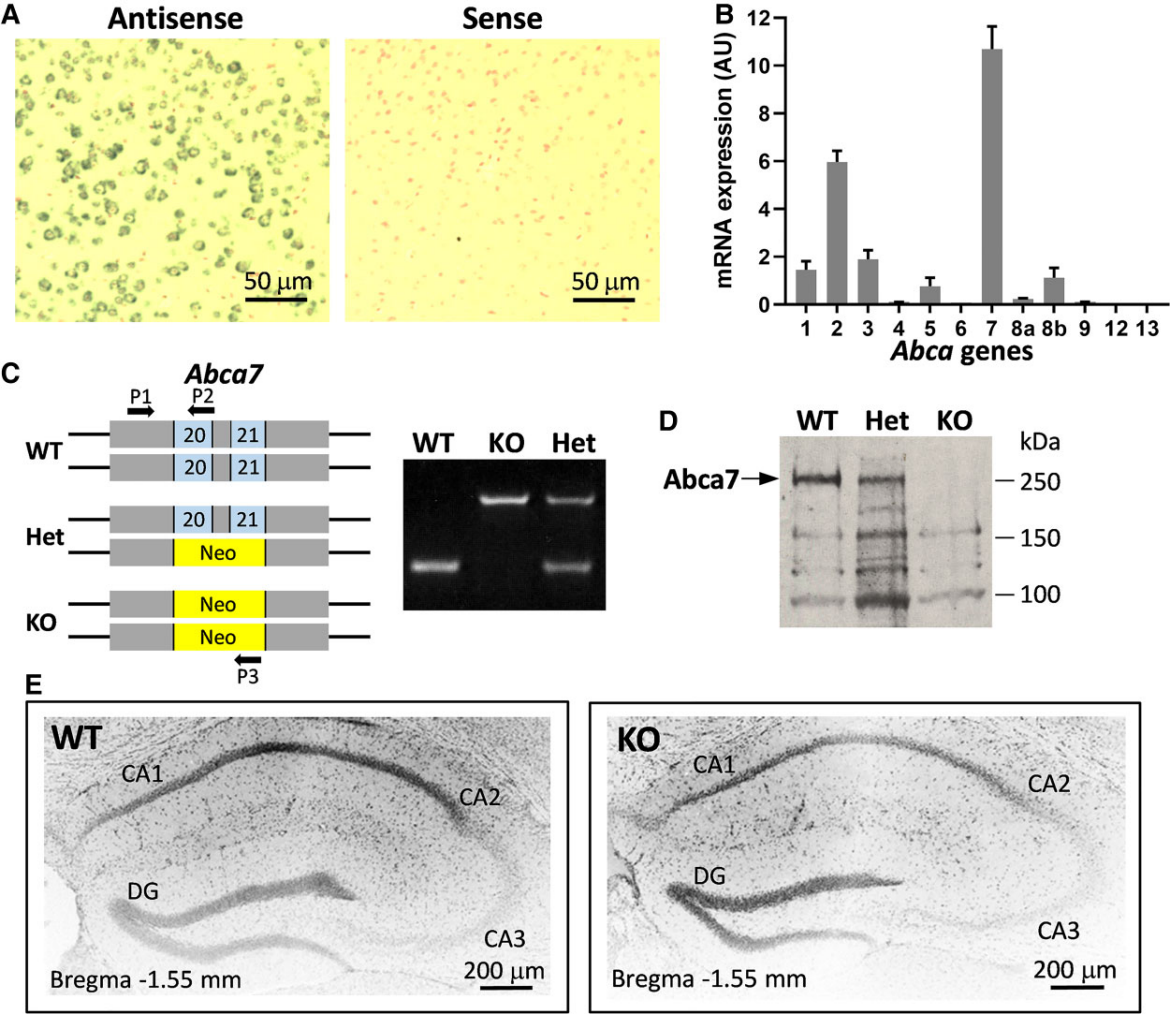
**Expression of Abca7 in the mouse brain**. (**A**) *In situ* hybridization of *Abca7* mRNA with digoxigenin-labelled antisense and sense probes and counterstained with nuclear Fast Red. (**B**) mRNA expression (Arbitrary Unit) of members of the *Abca* gene subfamily in the mouse brain (20 mg)— Abca1: 1.45, Abca2: 5.96, Abca3: 1.89, Abca4: 0.1, Abca5: 0.76, Abca6: 0.07, Abca7: 10.69, Abca8a: 0.22, Abca8b: 1.13, Abca9: 0.1, Abca12: 0.007, Abca13: 0.01. (**C**) Multiplex PCR genotyping of *Abca7* knock mouse with neomycin replacing *Abca7* exons 20 and 21. (**D**) Western blotting of Abca7 protein in the mouse brain. (**E**) Histology of the hippocampus of wild type and *Abca7* knock mouse stained with 4′,6-diamidino-2-phenylindole.

Firstly, we verified the strong expression of *Abca7* in the mouse brain (Fig. 1A). Of the 12 Abca subfamily members, Abca7 is expressed the strongest in the brain (Fig. 1B). We also verified the deletion of *Abca7* in the *Abca7* KO mice by PCR (Fig. 1C; Supplementary Fig. 1) and western blotting (Fig. 1D; Supplementary Fig. 2). These *Abca7* KO mice were generated from 19 backcrosses (i.e. 6 years of breeding) that absolutely minimizes heterologous genetic background. Histological analyses of the brain showed no overt difference between the KO and WT mice, and no formation of amyloid-β plaques in the KO mice (Fig. 1E) as expected since these are native mice that express only the endogenous mouse amyloid-β (not human amyloid-β transgenics).

**Figure 2:**
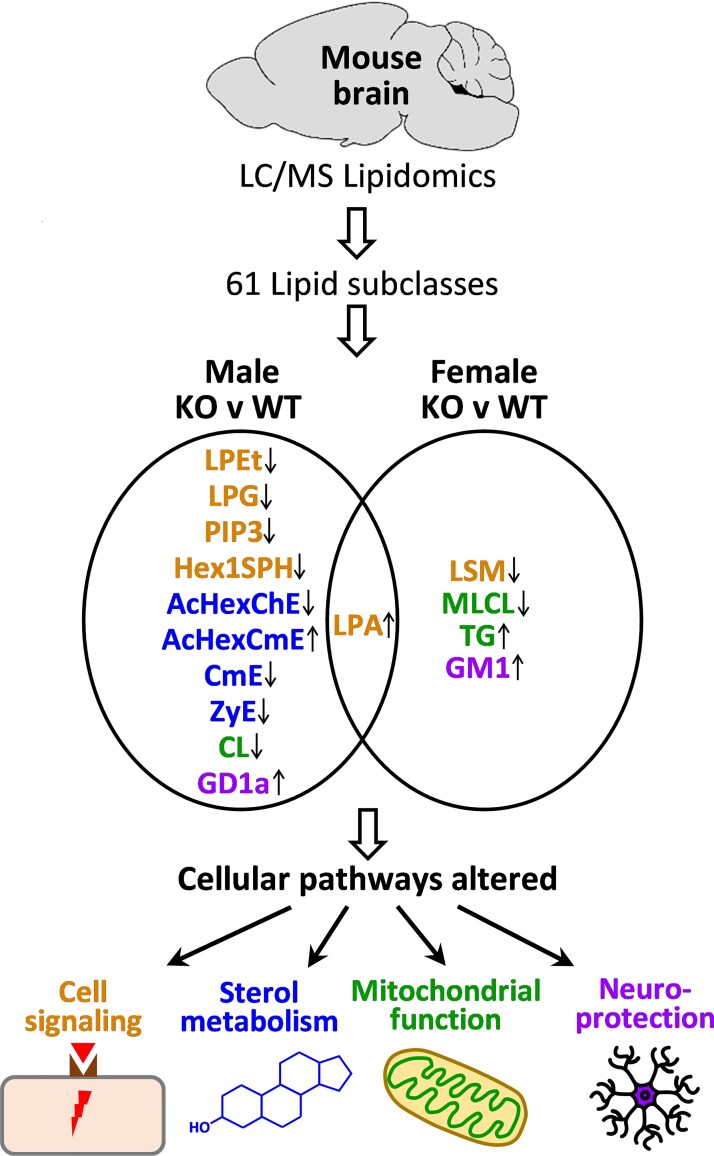
**The lipids that were altered in the *Abca7* knockout mouse brain and affected cellular pathways**. Sixty-one lipid subclasses were identified in the mouse brain and those that were altered in the male and female *Abca7* KO mice are shown. Up arrow indicates increased levels in the KO compared with WT mice. Down arrow indicates decreased levels in the KO compared with WT mice. Brown text indicates lipids altered in the cell signalling pathway. Blue text indicates lipids altered in sterol metabolism pathway. Green text indicates lipids altered in mitochondrial function pathway. Purple text indicates lipids altered in the neuroprotection pathway.

We then carried out quantitative discovery lipidomics using mass spectrometry on the brain of *Abca7* KO mice (*n* = 52) and WT littermates (*n* = 35). These are large cohorts in the context of animal studies that would allow uncovering subtle yet significant changes not possible with small cohorts. We identified 61 lipid subclasses in the mouse brain (Supplementary Table 1). The abundance of each lipid subclass was compared between the KO and WT animals for both sexes and those that were significantly altered are highlighted (Supplementary Table 1). In the male comparison, 11 lipid subclasses were significantly altered—acylhexosyl cholesterol ester (AcHexChE), acylhexosyl campesterol ester (AcHexCmE), cardiolipin (CL), campesterol ester (CmE), ganglioside disialo tetrahexosyl ceramide (GD1a), hexosyl sphingosine (Hex1SPH), lyso phosphatidic acid (LPA), lyso phosphatidylethanol (LPEt), lyso phosphatidylglycerol (LPG), phosphatidylinositol triphosphate (PIP3) and zymosterol ester (ZyE). Whereas, in the female comparison, only five lipid subclasses were significantly altered—ganglioside monosialo tetrahexosyl ceramide (GM1), LPA, lyso sphingomyelin (LSM), mono-lyso cardiolipin (MLCL) and triglyceride. The only lipid subclass that was significantly altered in both sexes was LPA. These lipids belong to cellular pathways that control cell signalling, sterol metabolism, mitochondrial function and neuroprotection (Fig. 2). All four pathways were affected in the male KO mice, whereas only three pathways (cell signalling, mitochondrial function and neuroprotection) were affected in the female KO mice.

### Analysis of App expression and processing in the *Abca7* KO mouse brain

**Figure 3:**
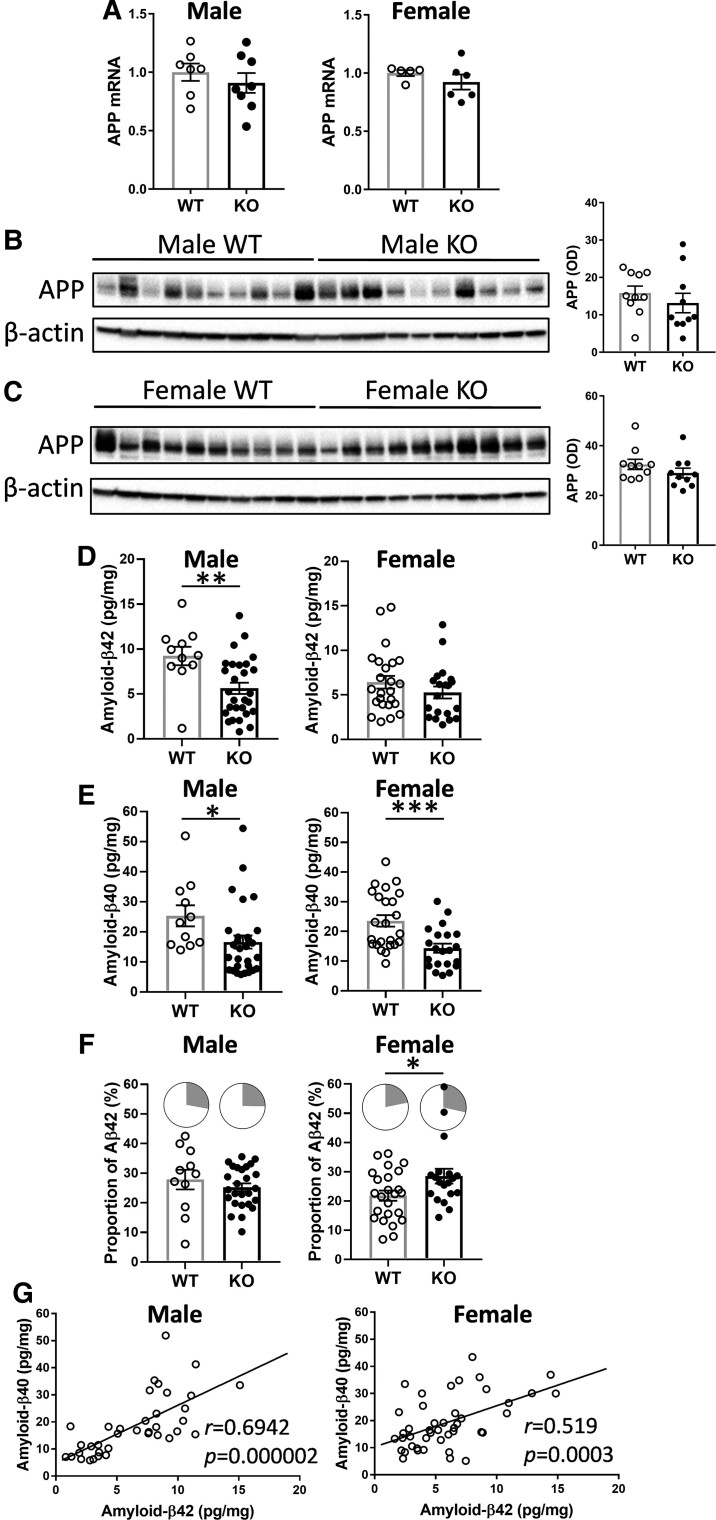
**Analysis of App expression in the *Abca7* KO mouse brain**. (**A**) *App* mRNA expression in male *Abca7* KO (*n* = 8) versus WT (*n* = 7) and female *Abca7* KO (*n* = 6) versus WT (*n* = 5), *t*-test, not significant. Each data point represents one animal. (**B**) App protein expression in male *Abca7* KO (*n* = 10) compared with male WT (*n* = 10) mice and optical density (OD) measurement of the protein bands normalized to β-actin; *t*-test, not significant. Each data point represents one animal. (**C**) App protein expression in female *Abca7* KO (*n* = 10) compared with female WT (*n* = 10) mice and OD measurement of the protein bands normalized to β-actin; *t*-test, not significant. Each data point represents one animal. (**D**) Amyloid-β42 levels in male *Abca7* KO (*n* = 31) versus WT (*n* = 11) and female *Abca7* KO (*n* = 21) versus WT (*n* = 24); *t*-test, **P < 0.005 *t* = 3.024. Each data point represents one animal. (**E**) Amyloid-β40 levels in male *Abca7* KO (*n* = 31) versus WT (*n* = 11) and female *Abca7* KO (*n* = 21) versus WT (*n* = 24); *t*-test, *P < 0.05 *t* = 2.117, ***P < 0.0005, *t* = 3.665. Each data point represents one animal. (**F**) Proportion of amyloid-β42 (grey sector) to total amyloid-β in male *Abca7* KO (*n* = 31) versus WT (*n* = 11) and female *Abca7* KO (*n* = 21) versus WT (*n* = 24); *t*-test, *P < 0.05 *t* = 2.230. Each data point represents one animal. (**G**) Correlation of amyloid-β42 and amyloid-β40 in male (*n* = 42) and female (*n* = 45) mouse brain.

In order to determine the relationship between lipid dysregulation and neuropathology, we carried out an analysis of amyloid precursor protein (App) expression and processing in the same brain tissue samples. Our *Abca7* KO mice express only the endogenous mouse *App* gene and produce only mouse amyloid-β at physiological levels. In contrast, most other mouse models, if not all, used in AD studies are transgenics that overexpress exogenous human mutant *APP* and produce extremely high levels of human amyloid-β in relatively young mice. Firstly, we measured *App* mRNA and protein expression by qPCR and western blotting. Both mRNA (Fig. 3A) and protein (Fig. 3B and C; Supplementary Figs. 3 and 4) were not altered with *Abca7* deletion in either sex.

Secondly, we measured amyloid-β42 and amyloid-β40 levels by ELISA. Amyloid-β42 levels were decreased only in the male KO mice (Fig. 3D), whereas amyloid-β40 levels were decreased in both male and female KO mice, although more significantly in the female KO mice (Fig. 3E). The proportion of amyloid-β42 (to total amyloid-β) was significantly increased only in the female KO mice (Fig. 3F). The two peptides were strongly correlated with each other in both male and female mice (Fig. 3G).

**Figure 4:**
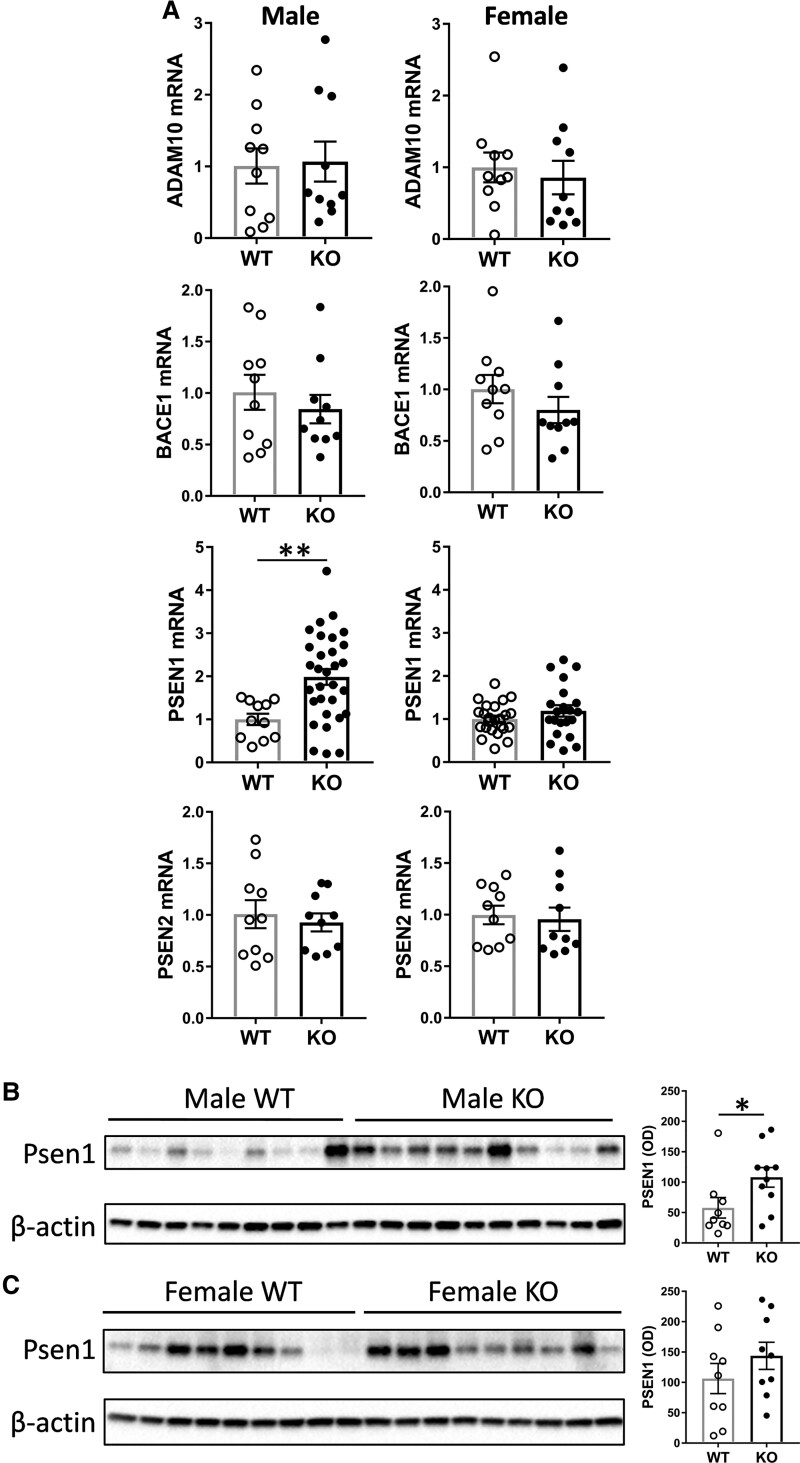
**Analysis of App processing genes in the *Abca7* KO mouse brain**. (**A**) mRNA expression of *Adam10*, *Bace1*, *Psen1* and *Psen2* in male *Abca7* KO (*n* = 10 or 31) versus WT (*n* = 10 or 11) and female *Abca7* KO (*n* = 10 or 21) versus WT (*n* = 10 or 24); *t*-test, **P < 0.005 *t* = 3.089. (**B**) Protein expression of Psen1 in male *Abca7* KO (*n* = 10) versus WT (*n* = 9) mice and optical density (OD) measurement of the protein bands normalized to β-actin; *t*-test, *P < 0.05 *t* = 2.148. (**C**) Protein expression of Psen1 in female *Abca7* KO (*n* = 9) versus WT (*n* = 9) mice and OD measurement of the protein bands normalized to β-actin; *t*-test, not significant.

Thirdly, we assessed the expression of the key genes, *Adam10*, *Bace1*, *Psen1* and *Psen2*, involved in App processing in the same tissue samples. *Psen1* was the only gene significantly altered, at both mRNA and protein levels, in the male KO mice (Fig. 4A and B; Supplementary Fig. 5). No genes were significantly altered in the female KO mice (Fig. 4A and C; Supplementary Fig. 6).

### Sex-specific differences in the effect of *Abca7* deletion on brain cholesterol

**Figure 5:**
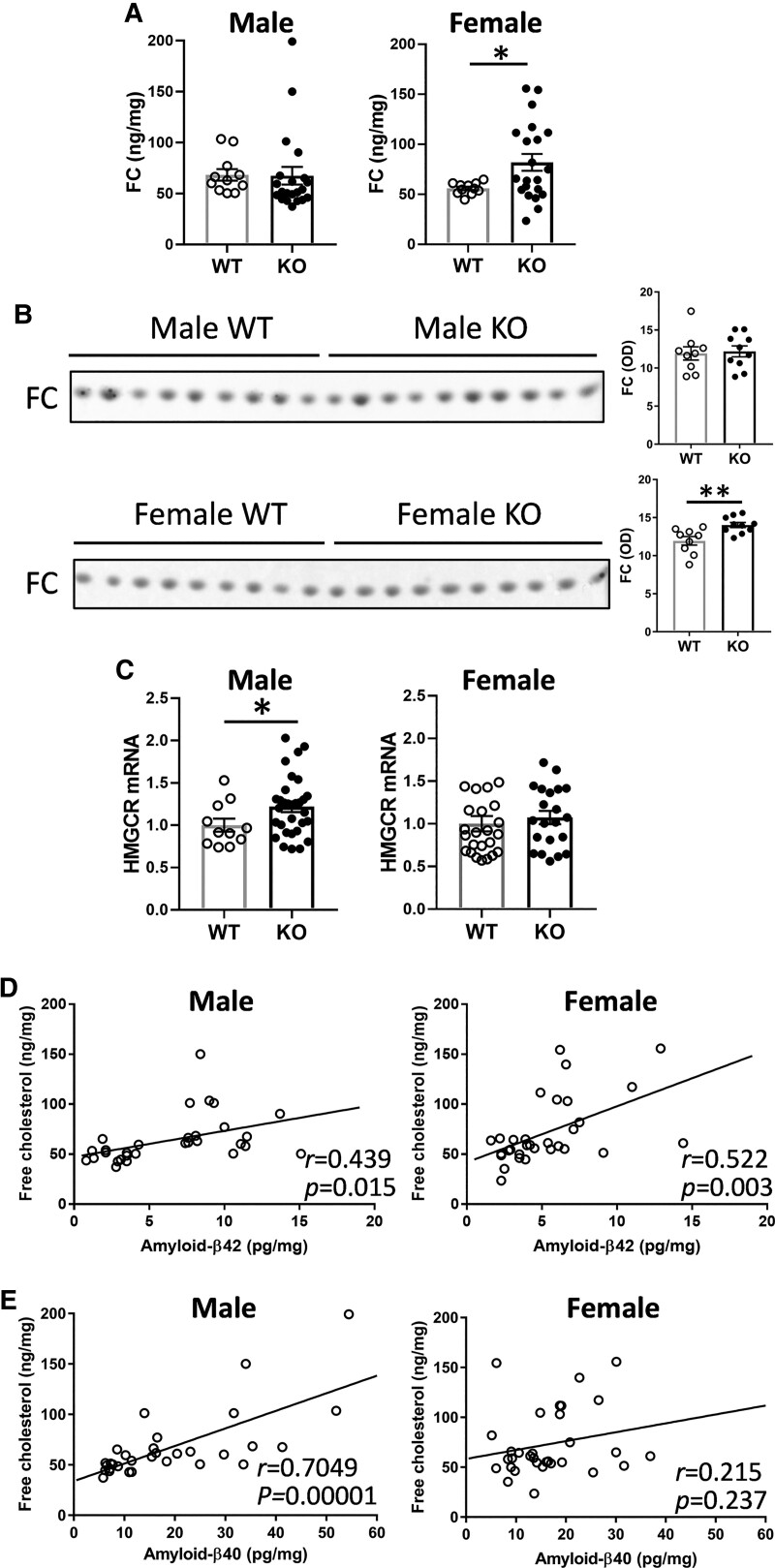
**Sex-specific impact of *Abca7* deletion on brain cholesterol**. (**A**) FC levels, as measured by enzymatic assay, in male *Abca7* KO (*n* = 21) versus WT (*n* = 11) and female *Abca7* KO (*n* = 21) versus WT (*n* = 11); *t*-test, *P < 0.05 *t* = 2.189. Each data point represents one animal. (**B**) FC levels, as measured by TLC, in male *Abca7* KO (*n* = 10) versus WT (*n* = 9) and female *Abca7* KO (*n* = 10) versus WT (*n* = 9) and optical density (OD) measurement of the FC staining; *t*-test, **P < 0.005 *t* = 3.256. Each data point represents one animal. (**C**) Transcription of 3-hydroxy-3-methyl-glutaryl-coenzyme A reductase (HMGCR) mRNA in male *Abca7* KO (*n* = 31) versus WT (*n* = 11) and female *Abca7* KO (*n* = 21) versus WT (*n* = 24); *t*-test, *P < 0.05 *t* = 2.014. Each data point represents one animal. (**D**) Correlation of FC and amyloid-β42 in male (*n* = 30) and female (*n* = 31) mouse brain. (**E**) Correlation of FC and amyloid-β40 in male (*n* = 31) and female (*n* = 32) mouse brain.

Of all brain lipids, cholesterol has been studied the most in the context of Alzheimer’s disease with elevated levels of cholesterol associated with an increased risk of Alzheimer’s disease.^32^ Cholesterol exists in two forms—FC and cholesterol ester (ChE). FC is a native cholesterol molecule, whereas ChE is a cholesterol molecule esterified with a fatty acid. In brain, the vast majority of cholesterol is in the form of FC, indicating that FC is of a greater relevance to brain function. We measured ChE as a part of the LC/MS lipidomics analysis and found that it was not altered with *Abca7* deletion in either sex (Supplementary Table 1). We then measured FC, in the same tissue samples, using a standard enzymatic essay (since un-esterified lipids cannot be measured by the LC/MS protocol). We found that FC levels were significantly increased only in the female KO mice (Fig. 5A). These results were verified with an alternative method of TLC (Fig. 5B; Supplementary Figs. 7 and 8). We also measured the transcription of 3-hydroxy-3-methyl-glutaryl-coenzyme A reductase (HMGCR), the rate-controlling enzyme in cholesterol synthesis, and found that it was upregulated only in the male KO mice (Fig. 5C). FC correlated to both amyloid-β42 and amyloid-β40 in male mice (Fig. 5D), whereas it correlated to only amyloid-β42 in female mice (Fig. 5E).

### Sex-specific differences in the effect of *Abca7* deletion on brain gangliosides

**Figure 6:**
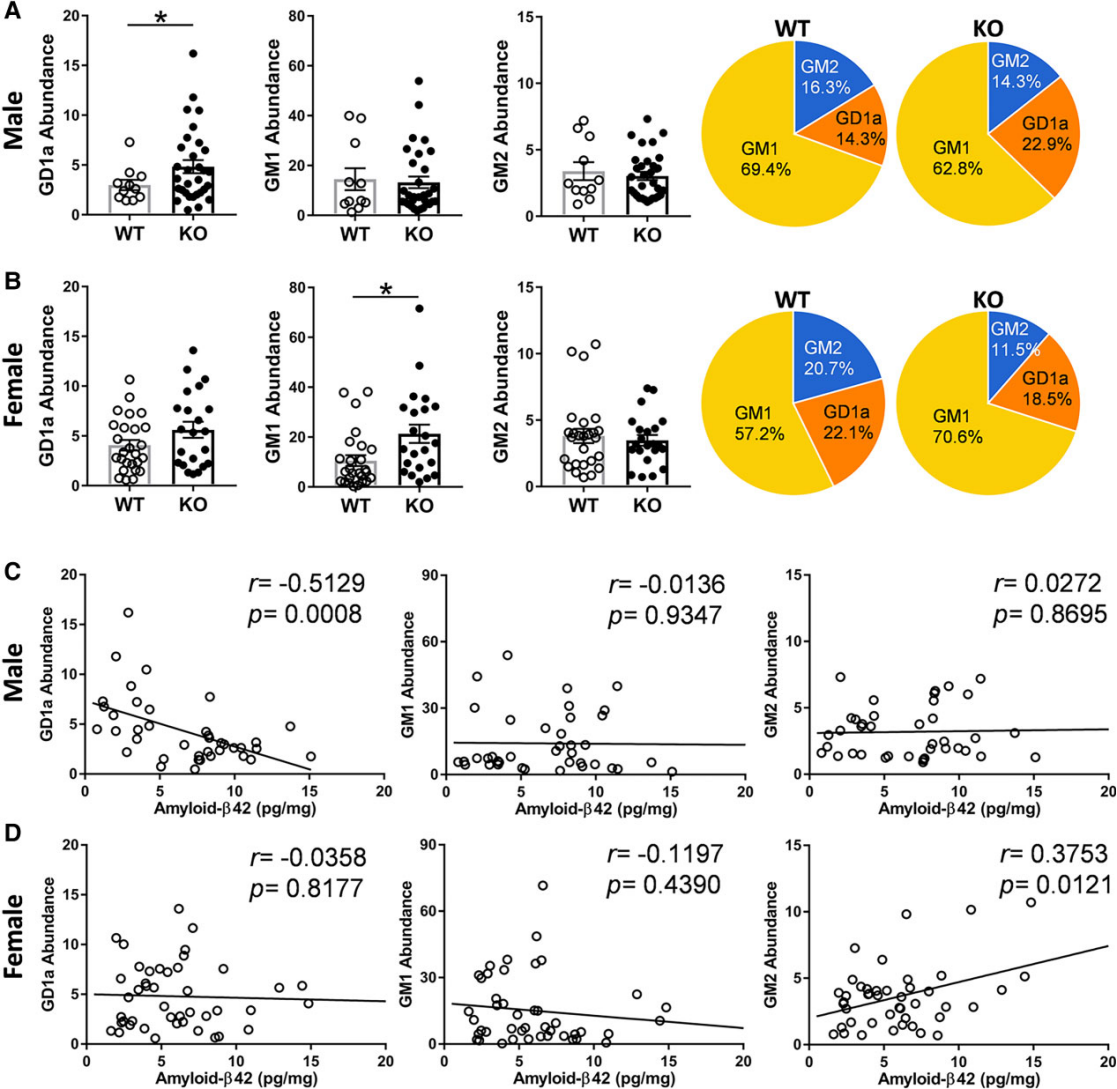
**Sex-specific impact of *Abca7* deletion on brain gangliosides**. (**A**) A comparison of gangliosides in male *Abca7* KO (*n* = 31) versus WT (*n* = 11) mice and distribution of gangliosides; *t*-test, *P < 0.05 *t* = 2.104. Each data point represents one animal. (**B**) A comparison of gangliosides in female *Abca7* KO (*n* = 21) versus WT (*n* = 24) mice and distribution of gangliosides; *t*-test, *P < 0.05 *t* = 2.609. Each data point represents one animal. (**C**) Correlation between gangliosides and amyloid-β42 in male (*n* = 39) mice. (**D**) Correlation between gangliosides and amyloid-β42 in female (*n* = 44) mice.

The importance of gangliosides in neuroprotection and in Alzheimer’s disease pathogenesis is starting to be recognized.^33^ Gangliosides are complex and unusual lipids in that they contain sugars (i.e. glucose and galactose) and sialic acid. They were originally identified in brain ganglion cells, but are highly abundant in neurons, and are enriched in plasma membrane lipid rafts.^34^ We identified three ganglioside subclasses (GD1a, GM1 and GM2) in the mouse brain (Supplementary Table 1) and found that GD1a was increased in the male KO mice (Fig. 6A), whereas GM1 was increased in the female KO mice (Fig. 6B). There were also sex-specific differences in the distribution of gangliosides (Figs. 6A and B). Interestingly, GD1a was inversely correlated with amyloid-β42 in male mice (Fig. 6C), whereas GM2 was correlated with amyloid-β42 in female mice (Fig. 6D).

### Correlation between brain lipids and amyloid-β42 levels

**Figure 7:**
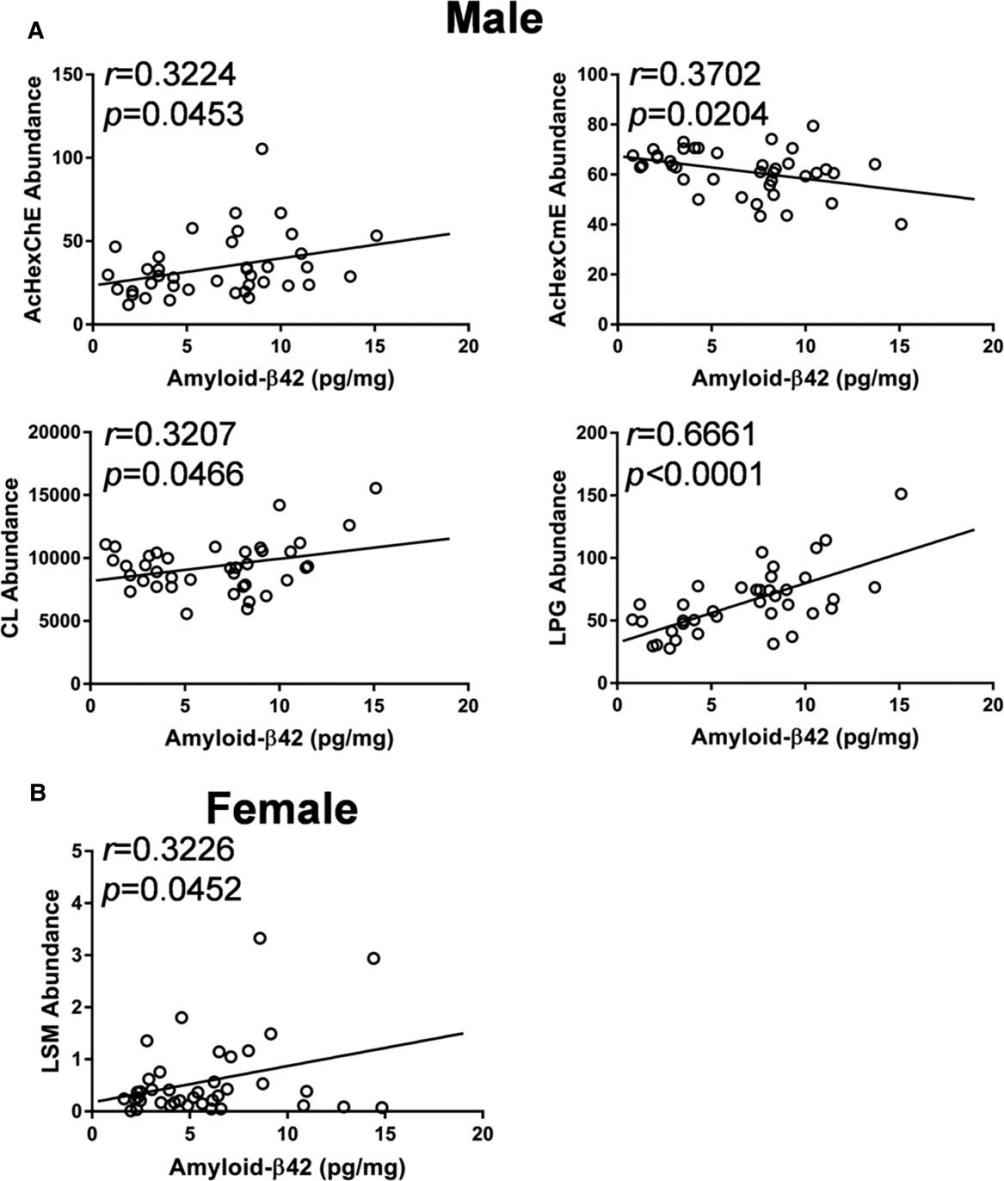
Other lipids that significantly correlated with amyloid-β42 in (**A**) male (*n* = 39) and (**B**) female (*n* = 44) mice.

Little is known about the link, if any, between most brain lipids and brain amyloid-β levels. We examined those lipids (other than cholesterol and gangliosides) that were altered in the KO mice (Supplementary Table 1). We found that AcHexChE, AcHexCmE, CL and LPG were significantly correlated to brain amyloid-β42 levels in male mice (Fig. 7A), whereas LSM was significantly correlated to brain amyloid-β42 levels in female mice (Fig. 7B).

## Discussion

Genetic variation in the *ABCA7* gene is regarded as the fourth-highest risk factor for Alzheimer’s disease. As a lipid transporter, ABCA7 regulates the translocation of cholesterol and phospholipids in the periphery. However, its role in lipid regulation in the brain is poorly understood and sex-specific differences in its cellular regulation of most lipids in the brain are unknown. Here, we performed a global assessment of brain lipids in male and female *Abca7* KO mice side-by-side. We identified 61 lipid subclasses in the mouse brain with *Abca7* deletion altering the levels of 11 in males compared with 5 in females. LPA is the only lipid subclass that was altered in the brains of both sexes. Correlations between brain amyloid-β and lipid levels also differed between male and female mice. Taken together, these results provide new knowledge on the brain lipids trafficked through the ABCA7 transporter and the potential basis for the sex-specific differences in the aetiology of Alzheimer’s disease.

Cholesterol has been implicated in Alzheimer’s disease pathogenesis in multiple pathways with elevated levels of cholesterol in the brain strongly associated with an increased risk of Alzheimer’s disease.^32,35–37^ In the present study, FC levels in the brain correlated with amyloid-β42 levels, with the FC levels elevated only in the brains of female and not male *Abca7* KO mice. In a feedback response to a high level of FC, HMGCR transcription would be reduced or maintained. Consistent with this, HMGCR transcription remained unchanged in the female mice. This suggests that pathophysiological changes associated with cholesterol are more severe in females compared with males. There were also sex-specific differences in other sterols. AcHexChE and ZyE, derivatives of cholesterol, and CmE and AcHexCmE, derivatives of campesterol (a plant sterol), were elevated only in male KO mice. It is unclear at this stage why these sterols are altered only in the male mice. We can only speculate that the levels of these sterols are somehow regulated by sex hormones (see below) or are altered as a result of some physiological or metabolic traits predominant in males. Brain AcHexChE levels correlated with the levels of amyloid-β42, whereas brain AcHexCmE levels inversely correlated with amyloid-β42 levels. Campesterol is believed to compete with cholesterol in the cholesterol absorption process,^38^ suggesting that exogenous (dietary) sterols can influence brain amyloid-β42 levels. The mice in our study were on the standard chow diet, which contains seeds and beans high in plant phytosterols. Another factor that needs to be taken into consideration is the body weight difference between male and female mice, with male mice being heavier, which may influence lipid metabolism. On this note, it will be important in future studies to compare lipid profiles of the brain with that of the periphery.

Another group of lipids important for neuronal and glial function are the gangliosides with ganglioside levels indicative of the structural integrity of neuronal and glial membranes.^39^ Gangliosides are complex-structured lipids enriched in cell membranes^40^ with GD1a one of the major gangliosides present in the brain.^41^ GD1a acts as a ligand for myelin-associated glycoprotein, inhibiting nerve regeneration.^42–44^ Previous studies show that GD1a levels correlated strongly with amyloid-β in Alzheimer’s disease mouse and cell models, and we found that brain GD1a levels inversely correlated with amyloid-β42, but only in male mice. Interestingly, brain GD1a levels were shown to be decreased in the substantia nigra of only male patients with Parkinson’s disease.^45^

Lysophospholipids (LPA, LPEt, LPG) are another group of lipids significantly altered in the *Abca7* KO mice. Lysophospholipids are derived from phospholipids, in which one of their two fatty acid chains is removed by hydrolysis. They are functionally different to phospholipids. Lysophospholipids are signalling molecules, whereas phospholipids serve as building blocks in cell membranes. Lysophospholipids typically bind to protein targets, such as receptors, kinases or phosphatases, which in turn activate specific cellular responses in a broad range of biological processes. LPA is such a signalling molecule that binds to G-protein coupled receptors on membranes that are distributed throughout the brain.^46^ Activation of LPA receptors initiates a number of cellular and developmental processes, such as survival, migration, adhesion, proliferation, differentiation, and myelination in neurons and oligodendrocytes in several regions of the brain, including the hippocampus and frontal cortex.^47–49^ The main LPA receptor is LPA1 and studies of *Lpa1*-null mice have shown a number of physiological and behavioural abnormalities relating to the hippocampus. These include reduction in hippocampal neurogenesis and neuronal maturation and survival,^50^ resulting in reduction in volume and neuronal density in the granular zone of the hippocampus.^51^ In terms of behaviour, *Lpa1*-null mice have profound cognitive deficits. *Lpa1*-null mice display impaired spatial memory, episodic-like memory tasks and contextual fear memory, as well altered exploration and increased anxiety-like behaviour.^52–54^ The level of LPA in the brain was increased in the *Abca7* KO mice in both sexes, possibly to compensate for the other detrimental lipid changes following *Abca7* deletion. Administration of LPA has been trialled in rodents to regulate LPA receptors with moderate success. In one study, infusion of LPA(18:1) into the hippocampus improved spatial memory consolidation.^55^

LPEt is derived from phosphatidylethanol, which is a phospholipid formed from ethanol. Phosphatidylethanol is thought to accumulate in the brain and contribute to alcohol intoxication.^56^ However, very little is known about the function of LPEt. It is similar in structure as phosphatidic acid and phosphatidylinositol 4,5-bisphosphate, suggesting a possible role as a signalling molecule. Also, very little is known about LPG in brain function. Unrelated to brain function, LPG was shown to induce chemotactic migration in endothelial cells^57^ and stimulate ERK and Akt signalling pathways in human ovarian cancer cells.^58^

Apart from lysophospholipids, three other signalling lipids (LSM, PIP3, Hex1SPH) were altered in the *Abca7* KO mice. LSM has a similar structure to sphingosine-1-phosphate (S1P) and can bind, at low affinity, to S1P receptors. It is involved in diverse biological processes in various cells and tissues, including inhibition of cell growth and cell proliferation.^59^ It also acts as an inhibitor of calmodulin, a highly prevalent intracellular calcium sensor.^60^ PIP3 is a potent signalling molecule that is important in anabolic signalling pathways required for cell growth and survival.^61^ It plays a role in hippocampal synaptic plasticity, maintenance of long-term potentiation and memory consolidation.^62,63^ Hex1SPH levels are altered in the brains of Gaucher disease, supposedly disrupting membrane raft structure and impairing cell signalling.^64^

CL is located in the inner mitochondrial membrane and is a key player in enzymatic processes that drive mitochondrial energy production.^65^ It is a gauge for mitochondrial integrity or energy output.^27^ MLCL is a derivative of CL, and has been extensively studied in the context of Barth syndrome. In Barth syndrome, MLCL levels are increased in the mitochondria, almost exclusively in males.^66^ In our study, MLCL levels were decreased only in the brains of female *Abca7* KO mice.

To understand the sex-specific differences in Alzheimer’s disease, much of the research focus has been on sex hormones, particularly oestrogen. In women, events that decrease lifetime exposure to oestrogen are associated with higher Alzheimer’s disease risk, whereas in men oestrogen does not exhibit age-related reduction nor is it associated with Alzheimer’s disease risk.^67^ Oestrogen levels in the post-mortem Alzheimer’s disease brain are lower compared with control brain.^68^ Reduction or loss of oestrogen levels increases the lifetime risk of dementia, as well as neuropathology and associated cognitive decline.^69,70^ During the menopause transition, where oestrogen levels are decreased, there is an increased vulnerability to Alzheimer’s disease.^71,72^ It appears that oestrogen is an agent that protects neurons from various molecules, including amyloid-β.^73–76^ The link between ABCA7 and oestrogen has not been explored in detail and the available information is very scant. In one study, it was shown that variation in the *ABCA7* gene is associated with an increased risk of Alzheimer’s disease in women with reduced levels of oestrogen at menopause.^77^ In an animal study, it was suggested that cessation of the oestrous cycling in female mice is a factor in the reduction in spatial reference memory performance in female *Abca7* KO mice.^24^ Much work is needed to understand the exact relationship between ABCA7 and oestrogen, and subsequent effects on Alzheimer’s disease pathogenesis. Given the fact that cholesterol, which is a substrate of the ABCA7 transporter, is a precursor molecule of oestrogen,^78^ it is conceivable that the function of ABCA7 is somehow related to oestrogen levels via cholesterol production or regulation. Consistent with this idea, *Abca7* deletion in mouse causes decreases in blood cholesterol only in female mice.^23^ Another aspect of oestrogen function related to Alzheimer’s disease is leptin, a hormone that modulates Alzheimer’s disease neuropathology, with decreases in blood leptin levels associated with increases in Alzheimer’s disease risk.^79–83^ Oestrogen induces leptin production^84^ and the two hormones are intrinsically linked to each other in synaptogenesis in the hypothalamic regions related to energy homeostasis.^85^*Abca7* deletion causes reduction in circulating leptin levels only in female mice,^86^ once again underscoring sex specificity in the function of ABCA7.

Both amyloid-β42 and amyloid-β40 levels were decreased in the brains of male *Abca7* KO mice, whereas only amyloid-β40 levels were decreased in the brains of female *Abca7* KO mice. The overall decrease in amyloid-β levels observed in our study is in contrast to typical increases reported in most other *Abca7* KO studies. A likely explanation for this difference is that our mice express only the endogenous mouse *App* gene and produce physiological levels of amyloid-β and no amyloid-β plaques are formed. In contrast, other *Abca7* KO mice are transgenics that typically express exogenous human mutant *APP* (e.g. APP-Swedish) and produce extremely high levels of human amyloid-β (not mouse amyloid-β) and form amyloid-β plaques in relatively young mice. These *Abca7* KO mice with human mutant *APP* transgenes have the expected significant pathological effects of increased amyloid-β processing. Consistent with our results, another study reported that endogenous mouse amyloid-β40 levels were significantly decreased in 24-week-old *Abca7* KO mice (an equal mix of male and female mice) compared with WT mice, although the brain levels were dependent on the age of the mice.^87^ At normal physiological levels, amyloid-β is thought to be involved in cholesterol trafficking in maintaining cholesterol homeostasis.^88^ Therefore, the impact of Abca7 on functional pathways, including App processing, is likely to be different in native mice compared with transgenic mice.

Another significant difference between our *Abca7* KO mice and others is that our mice have been backcrossed no <19 generations that absolutely minimizes heterologous genetic background, which is problematic for most KO mice that have been backcrossed, typically, only few generations. Furthermore, the vast majority of studies on *Abca7* KO mice, if not all, were based on small groups of mice usually of only a single sex. No direct comparisons were made between male and female mice. In contrast, our study is based on large numbers of mice, i.e. 35 WT and 52 KO mice, of both sexes, that provide tremendous statistical power.

Lipids constitute approximately two-thirds of brain tissues and are the building blocks for membranes. Much evidence shows that many lipids act as signalling molecules in numerous processes that determine the health of cells and tissues. Our unbiased lipidomic study has demonstrated that deletion of the *Abca7* gene causes lipid dysregulation that impacts on brain cellular processes associated with Alzheimer’s disease pathology in a sex-specific manner. Our new data provide insight into the underlying sex disparity in the aetiology of Alzheimer’s disease.

## Supplementary Material

fcac120_Supplementary_Data

## Abbreviations

ABCA7 =: ATP-binding cassette subfamily A member 7
AcHexChE =: acylhexosyl cholesterol ester
AcHexCmE =: acylhexosyl campesterol ester
APOE =: apolipoprotein E
APP =: amyloid precursor protein
ChE =: cholesterol ester
CL =: cardiolipin
CmE =: campesterol ester
FC =: free cholesterol
GD1a =: ganglioside disialo tetrahexosyl ceramide
GM1 =: ganglioside monosialo tetrahexosyl ceramide
GM2 =: ganglioside monosialo trihexosyl ceramide
HDL =: high-density lipoprotein
Hex1SPH =: hexosyl sphingosine
HMGCR =: 3-hydroxy-3-methyl-glutaryl-coenzyme A reductase
KO =: knockout
LPA =: lyso phosphatidic acid
LPEt =: lyso phosphatidylethanol
LPG =: lyso phosphatidylglycerol
LSM =: lyso sphingomyelin
MLCL =: mono-lyso cardiolipin
PIP3 =: phosphatidylinositol triphosphate
SNP =: single nucleotide polymorphism
WT =: wild type
ZyE =: zymosterol ester

## References

[1] Andersen K , Launer LJ, Dewey ME, et al Gender differences in the incidence of AD and vascular dementia: The EURODEM Studies. EURODEM Incidence Research Group. Neurology. 1999;53(9):1992–1997. 10599770 10.1212/wnl.53.9.1992

[2] Hebert LE , Weuve J, Scherr PA, Evans DA. Alzheimer disease in the United States (2010-2050) estimated using the 2010 census. Neurology. 2013;80(19):1778–1783. 23390181 10.1212/WNL.0b013e31828726f5 PMC3719424

[3] Seshadri S , Wolf PA, Beiser A, et al Lifetime risk of dementia and Alzheimer’s disease. The impact of mortality on risk estimates in the Framingham Study. Neurology. 1997;49(6):1498–1504. 9409336 10.1212/wnl.49.6.1498

[4] Chene G , Beiser A, Au R, et al Gender and incidence of dementia in the Framingham Heart Study from mid-adult life. Alzheimers Dement. 2015;11(3):310–320. 24418058 10.1016/j.jalz.2013.10.005 PMC4092061

[5] 2020 Alzheimer’s disease facts and figures. Alzheimers Dement. 2020;16:391–460. 10.1002/alz.12068 32157811

[6] Mathys H , Davila-Velderrain J, Peng Z, et al Single-cell transcriptomic analysis of Alzheimer’s disease. Nature. 2019;570(7761):332–337. 31042697 10.1038/s41586-019-1195-2 PMC6865822

[7] Farrer LA , Cupples LA, Haines JL, et al Effects of age, sex, and ethnicity on the association between apolipoprotein E genotype and Alzheimer disease. A meta-analysis. APOE and Alzheimer Disease Meta Analysis Consortium. JAMA. 1997;278(16):1349–1356. 9343467

[8] Bretsky PM , Buckwalter JG, Seeman TE, et al Evidence for an interaction between apolipoprotein E genotype, gender, and Alzheimer disease. Alzheimer Dis Assoc Disord. 1999;13(4):216–221. 10609670 10.1097/00002093-199910000-00007

[9] Payami H , Zareparsi S, Montee KR, et al Gender difference in apolipoprotein E-associated risk for familial Alzheimer disease: A possible clue to the higher incidence of Alzheimer disease in women. Am J Hum Genet. 1996;58(4):803–811. 8644745 PMC1914663

[10] Nazarian A , Yashin AI, Kulminski AM. Genome-wide analysis of genetic predisposition to Alzheimer’s disease and related sex disparities. Alzheimers Res Ther. 2019;11(1):5. 30636644 10.1186/s13195-018-0458-8 PMC6330399

[11] Carter CL , Resnick EM, Mallampalli M, Kalbarczyk A. Sex and gender differences in Alzheimer’s disease: Recommendations for future research. J Womens Health (Larchmt). 2012;21(10):1018–1023. 22917473 10.1089/jwh.2012.3789

[12] Henderson VW , Buckwalter JG. Cognitive deficits of men and women with Alzheimer’s disease. Neurology. 1994;44(1):90–96. 8290098 10.1212/wnl.44.1.90

[13] Hollingworth P , Harold D, Sims R, et al Common variants at ABCA7, MS4A6A/MS4A4E, EPHA1, CD33 and CD2AP are associated with Alzheimer’s disease. Nat Genet. 2011;43(5):429–435. 21460840 10.1038/ng.803 PMC3084173

[14] Steinberg S , Stefansson H, Jonsson T, et al Loss-of-function variants in ABCA7 confer risk of Alzheimer’s disease. Nat Genet. 2015;47(5):445–447. 25807283 10.1038/ng.3246

[15] Lambert JC , Ibrahim-Verbaas CA, Harold D, et al Meta-analysis of 74,046 individuals identifies 11 new susceptibility loci for Alzheimer’s disease. Nat Genet. 2013;45(12):1452–1458. 24162737 10.1038/ng.2802 PMC3896259

[16] Abe-Dohmae S , Ikeda Y, Matsuo M, et al Human ABCA7 supports apolipoprotein-mediated release of cellular cholesterol and phospholipid to generate high density lipoprotein. J Biol Chem. 2004;279(1):604–611. 14570867 10.1074/jbc.M309888200

[17] Brooks-Wilson A , Marcil M, Clee SM, et al Mutations in ABC1 in Tangier disease and familial high-density lipoprotein deficiency. Nat Genet. 1999;22(4):336–345. 10431236 10.1038/11905

[18] Fu Y , Hsiao JH, Paxinos G, Halliday GM, Kim WS. ABCA7 mediates phagocytic clearance of amyloid-beta in the brain. J Alzheimers Dis. 2016;54(2):569–584. 27472885 10.3233/JAD-160456

[19] Chan SL , Kim WS, Kwok JB, et al ATP-binding cassette transporter A7 regulates processing of amyloid precursor protein in vitro. J Neurochem. 2008;106(2):793–804. 18429932 10.1111/j.1471-4159.2008.05433.x

[20] Apostolova LG , Risacher SL, Duran T, et al Associations of the top 20 Alzheimer disease risk variants with brain amyloidosis. JAMA Neurol. 2018;75(3):328–341. 29340569 10.1001/jamaneurol.2017.4198 PMC5885860

[21] Nettiksimmons J , Tranah G, Evans DS, Yokoyama JS, Yaffe K. Gene-based aggregate SNP associations between candidate AD genes and cognitive decline. Age (Dordr). 2016;38(2):41. 27005436 10.1007/s11357-016-9885-2 PMC5005889

[22] Prokopenko D , Hecker J, Kirchner R, et al Identification of novel Alzheimer’s disease loci using sex-specific family-based association analysis of whole-genome sequence data. Sci Rep. 2020;10(1):5029. 32193444 10.1038/s41598-020-61883-6 PMC7081222

[23] Kim WS , Fitzgerald ML, Kang K, et al Abca7 null mice retain normal macrophage phosphatidylcholine and cholesterol efflux activity despite alterations in adipose mass and serum cholesterol levels. J Biol Chem. 2005;280(5):3989–3995. 15550377 10.1074/jbc.M412602200

[24] Logge W , Cheng D, Chesworth R, et al Role of Abca7 in mouse behaviours relevant to neurodegenerative diseases. PLoS One. 2012;7(9):e45959. 23029339 10.1371/journal.pone.0045959 PMC3454356

[25] Parlee SD , Lentz SI, Mori H, MacDougald OA. Quantifying size and number of adipocytes in adipose tissue. Methods Enzymol. 2014;537:93–122. 24480343 10.1016/B978-0-12-411619-1.00006-9 PMC4069255

[26] Galarraga M , Campion J, Munoz-Barrutia A, et al Adiposoft: Automated software for the analysis of white adipose tissue cellularity in histological sections. J Lipid Res. 2012;53(12):2791–2796. 22993232 10.1194/jlr.D023788 PMC3494244

[27] Phan K , He Y, Pickford R, et al Uncovering pathophysiological changes in frontotemporal dementia using serum lipids. Sci Rep. 2020;10(1):3640. 32107421 10.1038/s41598-020-60457-w PMC7046653

[28] He Y , Phan K, Bhatia S, et al Increased VLCFA-lipids and ELOVL4 underlie neurodegeneration in frontotemporal dementia. Sci Rep. 2021;11(1):21348. 34725421 10.1038/s41598-021-00870-x PMC8560873

[29] Kim WS , Jary E, Pickford R, et al Lipidomics analysis of behavioral variant frontotemporal dementia: A scope for biomarker development. Front Neurol. 2018;9:104. 29541056 10.3389/fneur.2018.00104 PMC5835505

[30] Cheng YS , Zheng Y, VanderGheynst JS. Rapid quantitative analysis of lipids using a colorimetric method in a microplate format. Lipids. 2011;46(1):95–103. 21069472 10.1007/s11745-010-3494-0

[31] Murphy KE , Cottle L, Gysbers AM, Cooper AA, Halliday GM. ATP13A2 (PARK9) protein levels are reduced in brain tissue of cases with Lewy bodies. Acta Neuropathol Commun. 2013;1:11. 24252509 10.1186/2051-5960-1-11 PMC4046687

[32] Shobab LA , Hsiung GY, Feldman HH. Cholesterol in Alzheimer’s disease. Lancet Neurol. 2005;4(12):841–852. 16297842 10.1016/S1474-4422(05)70248-9

[33] Blennow K , Davidsson P, Wallin A, et al Gangliosides in cerebrospinal fluid in ‘probable Alzheimer’s disease’. Arch Neurol. 1991;48(10):1032–1035. 1929894 10.1001/archneur.1991.00530220048018

[34] Dalton G , An SW, Al-Juboori SI, et al Soluble klotho binds monosialoganglioside to regulate membrane microdomains and growth factor signaling. Proc Natl Acad Sci U S A. 2017;114(4):752–757. 28069944 10.1073/pnas.1620301114 PMC5278494

[35] Liu CC , Liu CC, Kanekiyo T, Xu H, Bu G. Apolipoprotein E and Alzheimer disease: Risk, mechanisms and therapy. Nat Rev Neurol. 2013;9(2):106–118. 23296339 10.1038/nrneurol.2012.263 PMC3726719

[36] Matsuzaki T , Sasaki K, Hata J, et al Association of Alzheimer disease pathology with abnormal lipid metabolism: The Hisayama Study. Neurology. 2011;77(11):1068–1075. 21911734 10.1212/WNL.0b013e31822e145d

[37] Anstey KJ , Ashby-Mitchell K, Peters R. Updating the evidence on the association between serum cholesterol and risk of late-life dementia: Review and meta-analysis. J Alzheimers Dis. 2017;56(1):215–228. 27911314 10.3233/JAD-160826 PMC5240556

[38] Choudhary SP , Tran LS. Phytosterols: Perspectives in human nutrition and clinical therapy. Curr Med Chem. 2011;18(29):4557–4567. 21864283 10.2174/092986711797287593

[39] Seyfried TN , Yu RK. Ganglioside GD3: Structure, cellular distribution, and possible function. Mol Cell Biochem. 1985;68(1):3–10. 3903474 10.1007/BF00219383

[40] Svennerholm L , Bostrom K, Fredman P, Mansson JE, Rosengren B, Rynmark BM. Human brain gangliosides: Developmental changes from early fetal stage to advanced age. Biochim Biophys Acta. 1989;1005(2):109–117. 2775765 10.1016/0005-2760(89)90175-6

[41] Sturgill ER , Aoki K, Lopez PH, et al Biosynthesis of the major brain gangliosides GD1a and GT1b. Glycobiology. 2012;22(10):1289–1301. 22735313 10.1093/glycob/cws103 PMC3425327

[42] Alpaugh M , Galleguillos D, Forero J, et al Disease-modifying effects of ganglioside GM1 in Huntington’s disease models. EMBO Mol Med. 2017;9(11):1537–1557. 28993428 10.15252/emmm.201707763 PMC5666311

[43] Schneider JS . Altered expression of genes involved in ganglioside biosynthesis in substantia nigra neurons in Parkinson’s disease. PLoS One 2018;13(6):e0199189. 29902255 10.1371/journal.pone.0199189 PMC6002063

[44] Yamamoto N , Igbabvoa U, Shimada Y, et al Accelerated Abeta aggregation in the presence of GM1-ganglioside-accumulated synaptosomes of aged apoE4-knock-in mouse brain. FEBS Lett. 2004;569(1-3):135–139. 15225622 10.1016/j.febslet.2004.05.037

[45] Seyfried TN , Choi H, Chevalier A, Hogan D, Akgoc Z, Schneider JS. Sex-related abnormalities in substantia nigra lipids in Parkinson’s disease. ASN Neuro. 2018;10:1759091418781889. 10.1177/1759091418781889 PMC6024349 29932343

[46] Yung YC , Stoddard NC, Mirendil H, Chun J. Lysophosphatidic acid signaling in the nervous system. Neuron. 2015;85(4):669–682. 25695267 10.1016/j.neuron.2015.01.009 PMC4400838

[47] Aikawa S , Hashimoto T, Kano K, Aoki J. Lysophosphatidic acid as a lipid mediator with multiple biological actions. J Biochem. 2015;157(2):81–89. 25500504 10.1093/jb/mvu077

[48] Walker TL , Overall RW, Vogler S, et al Lysophosphatidic acid receptor is a functional marker of adult hippocampal precursor cells. Stem Cell Rep. 2016;6(4):552–565. 10.1016/j.stemcr.2016.03.002 PMC4834054 27050949

[49] Gonzalez de San Roman E , Manuel I, Ledent C, et al CB1 and LPA1 receptors relationship in the mouse central nervous system. Front Mol Neurosci. 2019;12:223. 31607860 10.3389/fnmol.2019.00223 PMC6761275

[50] Matas-Rico E , Garcia-Diaz B, Llebrez-Zayas P, et al Deletion of lysophosphatidic acid receptor LPA1 reduces neurogenesis in the mouse dentate gyrus. Mol Cell Neurosci. 2008;39(3):342–355. 18708146 10.1016/j.mcn.2008.07.014 PMC3667670

[51] Castilla-Ortega E , Hoyo-Becerra C, Pedraza C, et al Aggravation of chronic stress effects on hippocampal neurogenesis and spatial memory in LPA(1) receptor knockout mice. PLoS One. 2011;6(9):e25522. 21980482 10.1371/journal.pone.0025522 PMC3183048

[52] Santin LJ , Bilbao A, Pedraza C, et al Behavioral phenotype of maLPA1-null mice: Increased anxiety-like behavior and spatial memory deficits. Genes Brain Behav. 2009;8(8):772–784. 19689455 10.1111/j.1601-183X.2009.00524.x PMC4780438

[53] Castilla-Ortega E , Pedraza C, Chun J, de Fonseca FR, Estivill-Torrus G, Santin LJ. Hippocampal c-Fos activation in normal and LPA(1)-null mice after two object recognition tasks with different memory demands. Behav Brain Res. 2012;232(2):400–405. 22537775 10.1016/j.bbr.2012.04.018

[54] Pedraza C , Sanchez-Lopez J, Castilla-Ortega E, et al Fear extinction and acute stress reactivity reveal a role of LPA(1) receptor in regulating emotional-like behaviors. Brain Struct Funct. 2014;219(5):1659–1672. 23775489 10.1007/s00429-013-0592-9

[55] Dash PK , Orsi SA, Moody M, Moore AN. A role for hippocampal Rho-ROCK pathway in long-term spatial memory. Biochem Biophys Res Commun. 2004;322(3):893–898. 15336547 10.1016/j.bbrc.2004.08.004

[56] Chung HW , Petersen EN, Cabanos C, et al A molecular target for an alcohol chain-length cutoff. J Mol Biol. 2019;431(2):196–209. 30529033 10.1016/j.jmb.2018.11.028 PMC6360937

[57] Lee SY , Lee HY, Kim SD, Shim JW, Bae YS. Lysophosphatidylglycerol stimulates chemotactic migration and tube formation in human umbilical vein endothelial cells. Biochem Biophys Res Commun. 2007;363(3):490–494. 17888875 10.1016/j.bbrc.2007.08.194

[58] Park KS , Kim MK, Im DS, Bae YS. Effect of lysophosphatidylglycerol on several signaling molecules in OVCAR-3 human ovarian cancer cells: involvement of pertussis toxin-sensitive G-protein coupled receptor. Biochem Pharmacol. 2007;73(5):675–681. 17161826 10.1016/j.bcp.2006.11.010

[59] Xu Y , Zhu K, Hong G, et al Sphingosylphosphorylcholine is a ligand for ovarian cancer G-protein-coupled receptor 1. Nat Cell Biol. 2000;2(5):261–267. 10806476 10.1038/35010529

[60] Kovacs E , Liliom K. Sphingosylphosphorylcholine as a novel calmodulin inhibitor. Biochem J. 2008;410(2):427–437. 17979830 10.1042/BJ20071019

[61] Ma Q , Zhu C, Zhang W, et al Mitochondrial PIP3-binding protein FUNDC2 supports platelet survival via AKT signaling pathway. Cell Death Differ. 2019;26(2):321–331. 29786068 10.1038/s41418-018-0121-8 PMC6329745

[62] Arendt KL , Royo M, Fernandez-Monreal M, et al PIP3 controls synaptic function by maintaining AMPA receptor clustering at the postsynaptic membrane. Nat Neurosci. 2010;13(1):36–44. 20010819 10.1038/nn.2462 PMC2810846

[63] Xie MJ , Ishikawa Y, Yagi H, et al PIP3-Phldb2 is crucial for LTP regulating synaptic NMDA and AMPA receptor density and PSD95 turnover. Sci Rep. 2019;9(1):4305. 30867511 10.1038/s41598-019-40838-6 PMC6416313

[64] Schueler UH , Kolter T, Kaneski CR, et al Toxicity of glucosylsphingosine (glucopsychosine) to cultured neuronal cells: A model system for assessing neuronal damage in Gaucher disease type 2 and 3. Neurobiol Dis. 2003;14(3):595–601. 14678774 10.1016/j.nbd.2003.08.016

[65] Chicco AJ , Sparagna GC. Role of cardiolipin alterations in mitochondrial dysfunction and disease. Am J Physiol Cell Physiol. 2007;292(1):C33–C44. 16899548 10.1152/ajpcell.00243.2006

[66] Duncan AL . Monolysocardiolipin (MLCL) interactions with mitochondrial membrane proteins. Biochem Soc Trans. 2020;48(3):993–1004. 32453413 10.1042/BST20190932 PMC7329354

[67] Pike CJ . Sex and the development of Alzheimer’s disease. J Neurosci Res. 2017;95(1-2):671–680. 27870425 10.1002/jnr.23827 PMC5120614

[68] Yue X , Lu M, Lancaster T, et al Brain estrogen deficiency accelerates Abeta plaque formation in an Alzheimer’s disease animal model. Proc Natl Acad Sci U S A. 2005;102(52):19198–19203. 16365303 10.1073/pnas.0505203102 PMC1323154

[69] Bove R , Secor E, Chibnik LB, et al Age at surgical menopause influences cognitive decline and Alzheimer pathology in older women. Neurology. 2014;82(3):222–229. 24336141 10.1212/WNL.0000000000000033 PMC3902759

[70] Rocca WA , Bower JH, Maraganore DM, et al Increased risk of cognitive impairment or dementia in women who underwent oophorectomy before menopause. Neurology. 2007;69(11):1074–1083. 17761551 10.1212/01.wnl.0000276984.19542.e6

[71] Waring SC , Rocca WA, Petersen RC, O’Brien PC, Tangalos EG, Kokmen E. Postmenopausal estrogen replacement therapy and risk of AD: A population-based study. Neurology. 1999;52(5):965–970. 10102413 10.1212/wnl.52.5.965

[72] Zandi PP , Carlson MC, Plassman BL, et al Hormone replacement therapy and incidence of Alzheimer disease in older women: the Cache County Study. JAMA. 2002;288(17):2123–2129. 12413371 10.1001/jama.288.17.2123

[73] Woolley CS , McEwen BS. Estradiol mediates fluctuation in hippocampal synapse density during the estrous cycle in the adult rat. J Neurosci. 1992;12(7):2549–2554. 1613547 10.1523/JNEUROSCI.12-07-02549.1992 PMC6575846

[74] Inagaki T , Frankfurt M, Luine V. Estrogen-induced memory enhancements are blocked by acute bisphenol A in adult female rats: Role of dendritic spines. Endocrinology. 2012;153(7):3357–3367. 22569790 10.1210/en.2012-1121 PMC3380314

[75] Marin R , Guerra B, Hernandez-Jimenez JG, et al Estradiol prevents amyloid-beta peptide-induced cell death in a cholinergic cell line via modulation of a classical estrogen receptor. Neuroscience. 2003;121(4):917–926. 14580942 10.1016/s0306-4522(03)00464-0

[76] Nilsen J , Chen S, Irwin RW, Iwamoto S, Brinton RD. Estrogen protects neuronal cells from amyloid beta-induced apoptosis via regulation of mitochondrial proteins and function. BMC Neurosci. 2006;7:74. 17083736 10.1186/1471-2202-7-74 PMC1636062

[77] Ratnakumar A , Zimmerman SE, Jordan BA, Mar JC. Estrogen activates Alzheimer’s disease genes. Alzheimers Dement (N Y). 2019;5:906–917. 31890855 10.1016/j.trci.2019.09.004 PMC6926344

[78] Payne AH , Hales DB. Overview of steroidogenic enzymes in the pathway from cholesterol to active steroid hormones. Endocr Rev. 2004;25(6):947–970. 15583024 10.1210/er.2003-0030

[79] Greco SJ , Bryan KJ, Sarkar S, et al Leptin reduces pathology and improves memory in a transgenic mouse model of Alzheimer’s disease. J Alzheimers Dis. 2010;19(4):1155–1167. 20308782 10.3233/JAD-2010-1308 PMC2862270

[80] Beccano-Kelly D , Harvey J. Leptin: a novel therapeutic target in Alzheimer’s disease? Int J Alzheimers Dis. 2012;2012:594137. 22254146 10.1155/2012/594137 PMC3255100

[81] Fewlass DC , Noboa K, Pi-Sunyer FX, Johnston JM, Yan SD, Tezapsidis N. Obesity-related leptin regulates Alzheimer’s Abeta. FASEB J. 2004;18(15):1870–1878. 15576490 10.1096/fj.04-2572com

[82] Power DA , Noel J, Collins R, O’Neill D. Circulating leptin levels and weight loss in Alzheimer’s disease patients. Dement Geriatr Cogn Disord. 2001;12(2):167–170. 11173891 10.1159/000051252

[83] Holden KF , Lindquist K, Tylavsky FA, Rosano C, Harris TB, Yaffe K. Serum leptin level and cognition in the elderly: Findings from the Health ABC Study. Neurobiol Aging. 2009;30(9):1483–1489. 18358569 10.1016/j.neurobiolaging.2007.11.024 PMC5278645

[84] Ahima RS , Flier JS. Leptin. Annu Rev Physiol. 2000;62:413–437. 10845097 10.1146/annurev.physiol.62.1.413

[85] Gao Q , Horvath TL. Cross-talk between estrogen and leptin signaling in the hypothalamus. Am J Physiol Endocrinol Metab. 2008;294(5):E817- E826. 18334610 10.1152/ajpendo.00733.2007

[86] Bhatia S , Fu Y, Hsiao JT, Halliday GM, Kim WS. Deletion of Alzheimer’s disease risk gene ABCA7 alters white adipose tissue development and leptin levels. J Alzheimers Dis Rep. 2017;1(1):237–247. 30480241 10.3233/ADR-170029 PMC6159609

[87] Satoh K , Abe-Dohmae S, Yokoyama S, St George-Hyslop P, Fraser PE. ATP-binding cassette transporter A7 (ABCA7) loss of function alters Alzheimer amyloid processing. J Biol Chem. 2015;290(40):24152–24165. 26260791 10.1074/jbc.M115.655076 PMC4591804

[88] Yao ZX , Papadopoulos V. Function of beta-amyloid in cholesterol transport: A lead to neurotoxicity. FASEB J. 2002;16(12):1677–1679. 12206998 10.1096/fj.02-0285fje

