# Supplementary material for: Sex-specific lipid dysregulation in the Abca7 knockout mouse brain
Source: Brain Commun. 2022 May 11;4(3):fcac120. doi: 10.1093/braincomms/fcac120 (PMC9127619; doi:10.1093/braincomms/fcac120)
Supplement: fcac120_Supplementary_Data [file fcac120_supplementary_data.zip › Supplementary Table 1.pdf]

Supplementary Table 1 Quantitative discovery lipidomics of *Abca7* knockout and wild type mouse brain. Blue text indicates lipids that were significantly altered in male *Abca7* KO mice compared to male WT mice; purple text indicates lipids that were significantly altered in female *Abca7* KO mice compared to female WT mice; and orange text indicates lipids that were significantly altered in both sexes.

Lipid subclass	Symbol	Male WT	Male KO	P value	Female WT	Female KO	P value
N-Acylethanolamine	AEA	13.41±3.09	10.41±1.84	0.4103	11.49±2.41	8.33±2.61	0.3787
Acyl Carnitine	AcCa	7457.29±913.77	5931.76±544.32	0.1593	6949.00±767.37	6432.82±834.22	0.6510
**Acyl Hexosyl Cholesterol ester**	**AcHexChE**	**46.46±5.21**	**30.72±3.10**	**0.0132**	33.83±2.59	26.29±2.82	0.0551
**Acyl Hexosyl Campesterol ester**	**AcHexCmE**	**56.55±2.60**	**63.13±1.55**	**0.0357**	60.82±1.84	59.67±2.00	0.6748
Acyl Hexosyl Zymosterol ester	AcHexZyE	2086.77±93.51	2017.71±55.70	0.5294	1994.89±69.42	1835.84±75.46	0.1277
Bis-methyl phosphatidic acid	BisMePA	113477.37±3593.99	117970.47±2140.88	0.2892	112621.81±2621.57	112618.23±2849.94	0.9993
Bis-methyl phosphatidyl ethanolamine	BisMePE	1593.92±121.70	1606.06±72.49	0.9321	1443.89±67.07	1487.78±72.91	0.6598
**Cardiolipin**	**CL**	**10487.99±577.39**	**8871.64±343.94**	**0.0209**	9390.22±328.54	9210.48±357.16	0.7128
Ceramide	Cer	6847.94±762.67	6558.55±454.31	0.7461	6031.35±379.76	6971.69±412.84	0.1005
Dihexosyl N-acetylhexosyl ceramide	CerG2GNAc1	28.27±3.08	28.34±1.83	0.9842	30.73±3.08	28.52±3.35	0.6289
Trihexosyl di-N-acetylhexosyl ceramide	CerG3GNAc2	0.30±0.02	0.27±0.01	0.3158	0.30±0.02	0.26±0.02	0.2127
Ceramide phosphate	CerP	1.49±0.46	1.68±0.27	0.7173	2.19±0.30	2.16±0.32	0.9607
Ceramide phosphoethanolamine	CerPE	50.62±7.22	49.49±4.30	0.8936	52.31±5.05	40.93±5.49	0.1343
Cholesterol ester	ChE	161.18±40.95	135.57±24.80	0.5958	133.96±22.68	138.69±24.65	0.8885
**Campesterol ester**	**CmE**	**5.43±0.54**	**3.43±0.32**	**0.0030**	4.54±0.70	5.22±0.76	0.5131
Cyclic phosphatidic acid	cPA	0.18±0.06	0.15±0.03	0.6627	0.18±0.03	0.12±0.04	0.2173
Diglyceride	DG	11556.50±939.43	9782.08±559.60	0.1125	12098.97±1049.94	9648.50±1141.40	0.1209
Dimethyl phosphatidylethanolamine	dMePE	25115.52±1715.32	28079.08±1021.79	0.1456	30289.86±1458.99	28776.36±1586.09	0.4860
Fatty acid	FA	680.95±73.56	663.63±43.82	0.8407	682.60±49.27	615.24±53.56	0.3594
**Ganglioside, disialo tetrahexosyl ceramide**	**GD1a**	**2.99±1.71**	**4.83±3.67**	**0.0344**	4.07±2.71	5.62±3.76	0.1156
**Ganglioside, monosialo tetrahexosyl ceramide**	**GM1**	14.51±14.52	13.23±13.00	0.8002	**10.53±11.12**	**21.28±17.18**	**0.0165**
Ganglioside, monosialo trihexosyl ceramide	GM2	3.40±0.57	3.03±0.34	0.5806	3.81±0.47	3.48±0.51	0.6349
Hexosyl ceramide	Hex1Cer	151001.83±7282.84	156011.46±4338.27	0.5579	151323.99±6412.17	154591.90±6970.77	0.7316
Dihexosyl ceramide	Hex2Cer	82987.70±4823.72	80788.81±2873.41	0.6974	71690.61±3190.58	73549.56±3468.53	0.6951
**Hexosyl sphingosine**	**Hex1SPH**	**121.06±27.12**	**14.23±16.15**	**0.0016**	15.17±20.50	56.91±22.29	0.1748
**Lyso phosphatidic acid**	**LPA**	**618.98±364.03**	**1610.83±216.84**	**0.0243**	**1082.01±446.35**	**2947.65±485.24**	**0.0069**
Lyso phosphatidylcholine	LPC	15159.29±1099.37	13380.97±654.88	0.1723	16522.17±1097.79	14944.26±1193.43	0.3356
Lyso phosphatidylethanolamine	LPE	9810.68±773.56	8000.19±460.79	0.0511	8379.00±617.24	9108.79±671.01	0.4276
**Lyso phosphatidylethanol**	**LPEt**	**7.65±1.50**	**2.69±0.89**	**0.0070**	5.51±0.98	2.85±1.07	0.0731
**Lyso phosphatidylglycerol**	**LPG**	**83.28±7.08**	**56.67±4.22**	**0.0025**	61.96±4.34	60.49±4.72	0.8190
Lyso phosphatidylinositol	LPI	75.80±14.00	83.95±8.34	0.6198	84.97±8.61	69.22±9.36	0.2220
Lyso phosphatidylserine	LPS	67.23±7.29	57.10±4.34	0.2396	54.21±2.97	54.98±3.23	0.8604
**Lyso sphingomyelin**	**LSM**	0.54±0.13	0.58±0.08	0.7998	**0.73±0.13**	**0.33±0.14**	**0.0420**
Lyso dimethyl phosphatidyl ethanolamine	LdMePE	713.22±92.01	606.70±54.81	0.3259	728.17±71.63	636.13±77.87	0.3888
Monoglyceride	MG	624.66±1095.00	1683.01±652.27	0.4113	655.38±81.84	502.96±88.97	0.2137
Monogalactosyl diacylglycerol	MGDG	3626.04±227.93	3592.99±135.77	0.9015	3495.48±136.80	3380.33±148.72	0.5716
Monogalactosyl monoacylglycerol	MGMG	407.06±39.29	383.90±23.40	0.6153	417.17±30.06	426.22±32.68	0.8394
**Mono-lyso cardiolipin**	**MLCL**	8.46±0.81	7.98±0.48	0.6083	**9.82±0.58**	**8.03±0.63**	**0.0416**
Methyl phosphatidylcholine	MePC	142302.60±22591.90	159370.64±13457.62	0.5200	164587.70±16592.58	154768.97±18038.03	0.6906
O-Acyl-(gamma-hydroxy) fatty acid	OAHFA	31.01±4.92	31.33±2.93	0.9558	35.25±4.25	28.33±4.62	0.2764
Phosphatidic acid	PA	195371.93±115213.44	169697.37±68630.74	0.8491	278000.17±133331.97	370929.12±144947.13	0.6393
Platelet-activating factor	PAF	1274.53±148.30	1198.37±88.34	0.6614	1445.82±152.33	1175.03±165.60	0.2350
Phosphatidylcholine	PC	678420.86±44613.55	761885.88±26575.55	0.1159	761060.04±56097.00	822022.68±60983.87	0.4656
Phosphatidylethanolamine	PE	262768.96±26420.92	316801.02±15738.50	0.0866	324472.27±20039.15	296349.10±21784.86	0.3470
Phosphatidylethanol	PEt	3817.36±526.48	4056.08±313.62	0.6989	4578.44±663.04	3878.80±720.80	0.4786
Phosphatidylglycerol	PG	16588.43±1171.75	16746.90±697.99	0.9081	19274.71±1564.61	17487.83±1700.91	0.4434
Sphingomyelin, phytosphingosine	phSM	325.24±30.31	326.13±18.05	0.9798	316.17±16.03	312.04±17.43	0.8622
Phosphatidylinositol	PI	157420.71±30663.71	163870.48±18265.86	0.8575	175056.93±19881.48	158983.77±21613.44	0.5868
Phosphatidylinositol phosphate	PIP	10.10±2.93	11.39±1.75	0.7063	10.31±1.82	9.26±1.98	0.6992
Phosphatidylinositol diphosphate	PIP2	4.37±0.33	4.50±0.20	0.7391	4.39±0.23	3.97±0.25	0.2217
**Phosphatidylinositol triphosphate**	**PIP3**	**5.96±0.34**	**5.14±0.20**	**0.0453**	5.10±0.22	4.65±0.24	0.1697
Phosphatidylmethanol	PMe	1962.41±243.65	1971.11±145.14	0.9757	2052.75±135.85	1924.89±147.69	0.5272
Phosphatidylserine	PS	165558.85±23526.92	135767.28±14014.59	0.2832	172429.86±19247.81	187610.74±20924.58	0.5959
Sphingomyelin	SM	85719.73±19159.23	99521.49±11412.84	0.5395	111754.95±22128.06	136042.77±24055.74	0.4612
Sphingosine bases	SPH	529.10±28.73	509.75±17.11	0.5660	551.05±28.52	478.70±31.00	0.0926
Sphingosine phosphate	SPHP	4.72±3.69	8.44±2.20	0.3913	5.84±1.81	5.15±1.97	0.7994
Sulfatide (galactosyl cer sulfate)	ST	5202.50±400.22	5040.93±238.40	0.7305	4575.47±190.05	4695.62±206.61	0.6707
Stigmasterol ester	StE	3.98±1.70	3.94±1.01	0.9868	3.66±0.78	3.33±0.85	0.7742
**Triglyceride**	**TG**	69456.21±4126.76	67009.35±2458.24	0.6133	**67803.06±3289.34**	**79578.19±3575.88**	**0.0194**
Wax esters (fatty acid esters)	WE	263.05±40.00	223.79±23.83	0.4041	229.76±39.00	207.04±42.40	0.6952
**Zymosterol ester**	**ZyE**	**3.08±0.34**	**2.13±0.20**	**0.0190**	3.01±0.24	2.38±0.26	0.0809
