# Supplementary material for: Sex-specific lipid dysregulation in the Abca7 knockout mouse brain
Source: Brain Commun. 2022 May 11;4(3):fcac120. doi: 10.1093/braincomms/fcac120 (PMC9127619; doi:10.1093/braincomms/fcac120)

Supplementary Fig. 1 PCR genotyping of Abca7 knock mouse

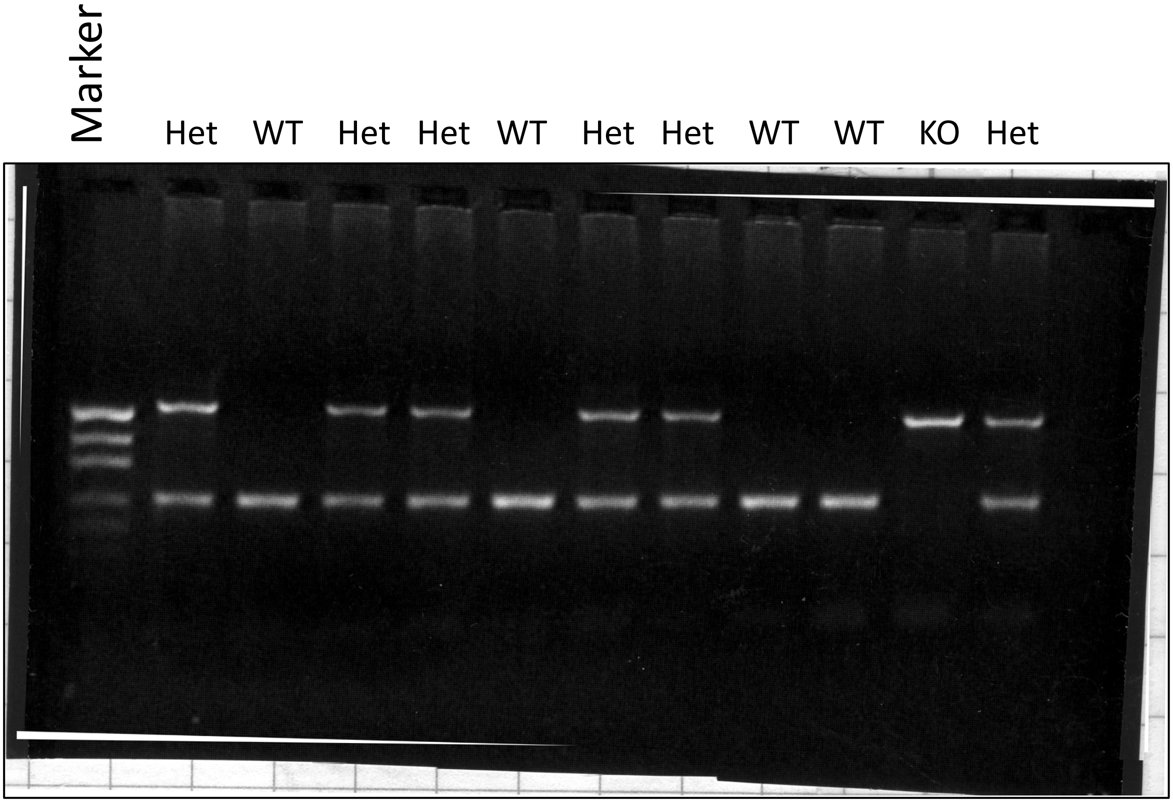

Supplementary Fig. 2 Western blotting of Abca7 protein in the mouse brain

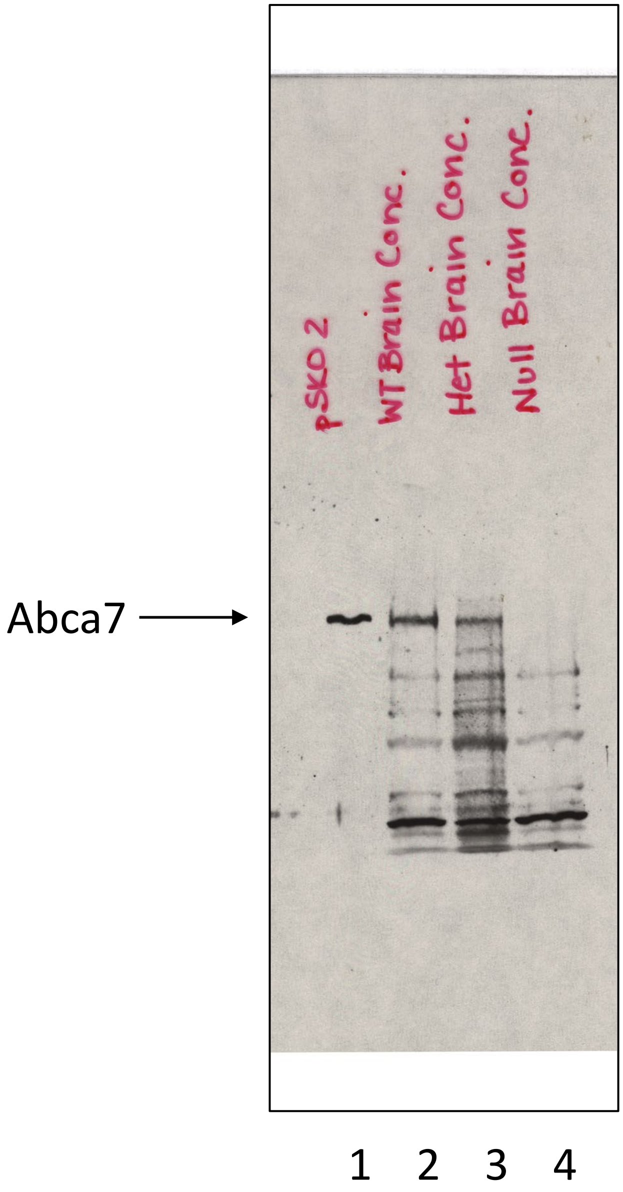

Legend

1. Positive Abca7 protein control
2. Wild type mouse brain
3. Heterozygous mouse brain
4. Abca7 knockout mouse brain

Supplementary Fig. 3 Expression of APP and β-actin in male brain

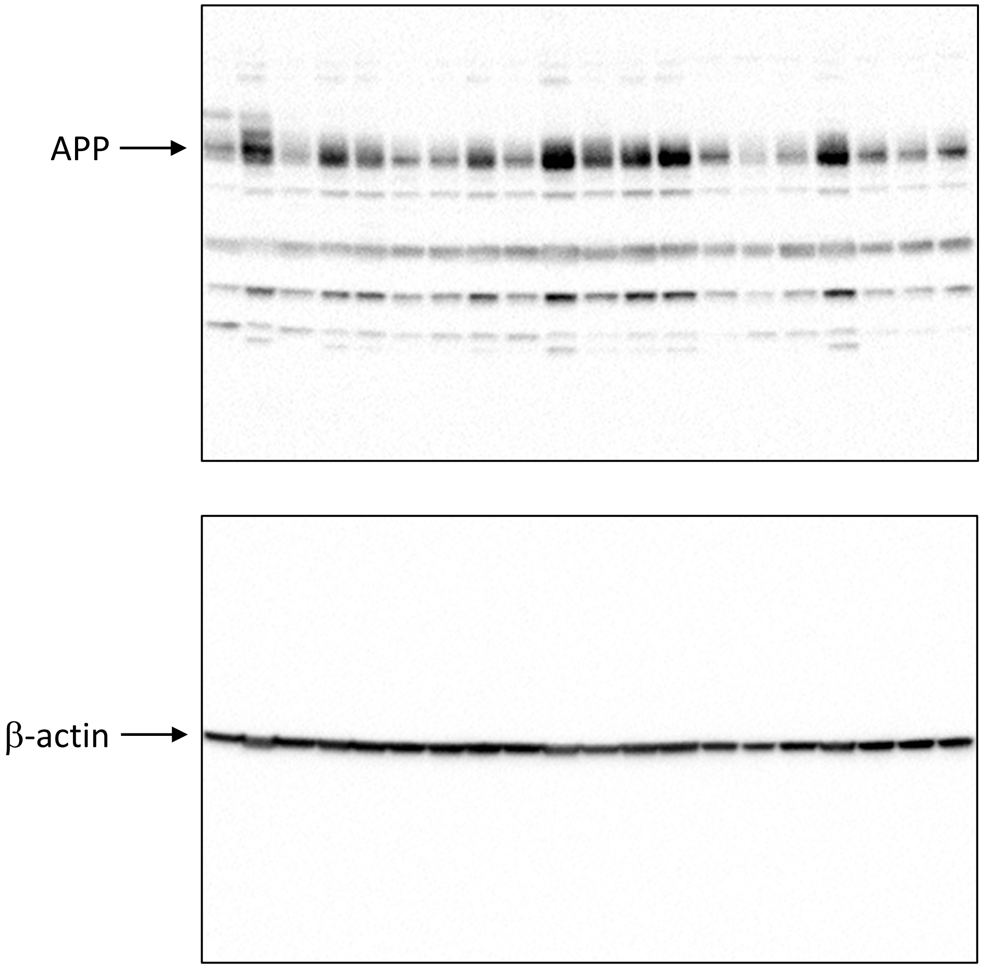

Supplementary Fig. 4 Expression of APP and β-actin in female brain

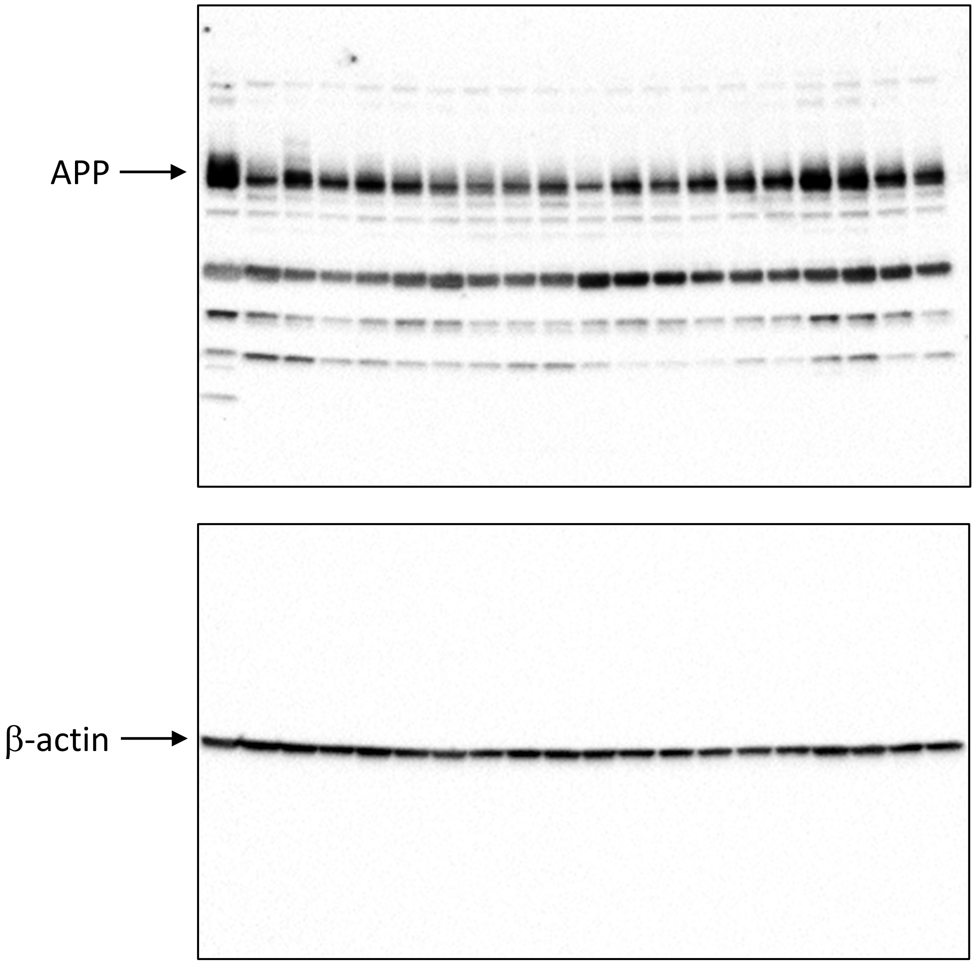

Supplementary Fig. 5 Expression of Psen1 and β-actin in male brain

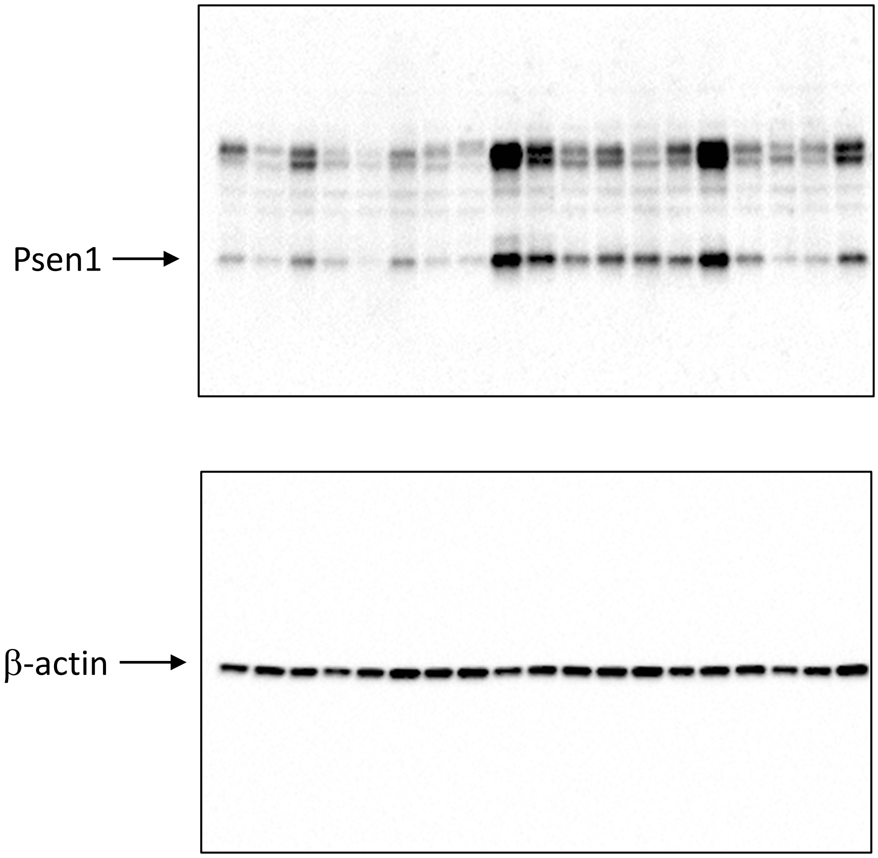

Supplementary Fig. 6 Expression of Psen1 and β-actin in female brain

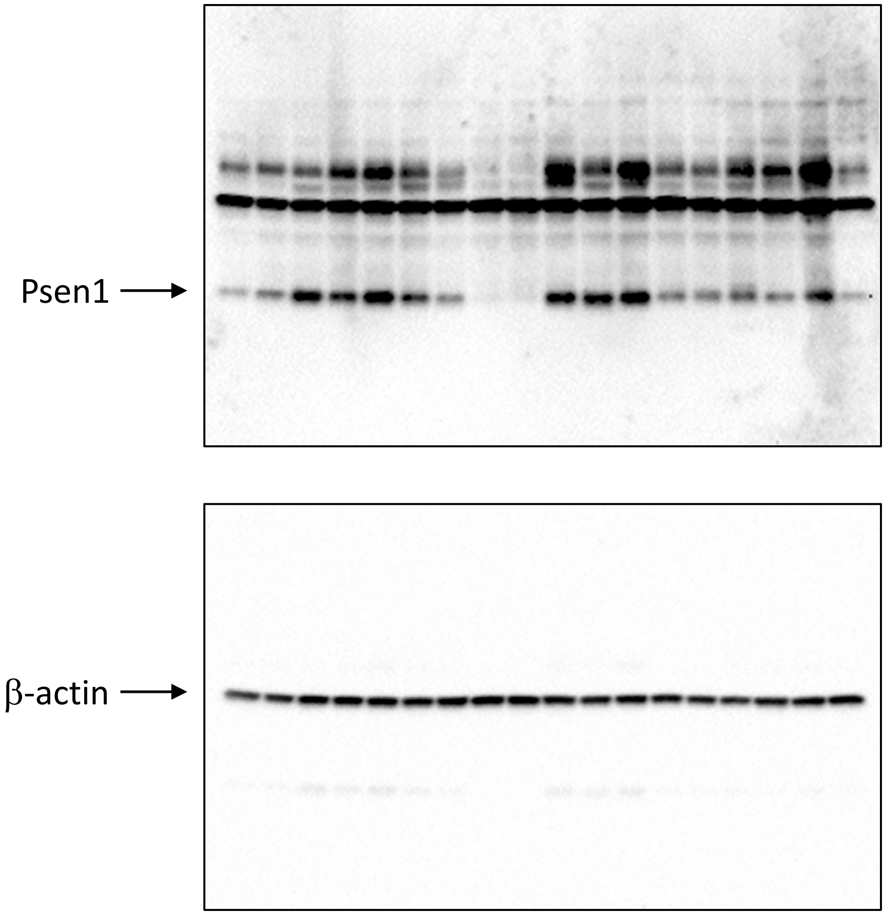

Supplementary Fig. 7 Thin layer chromatogram of male brain

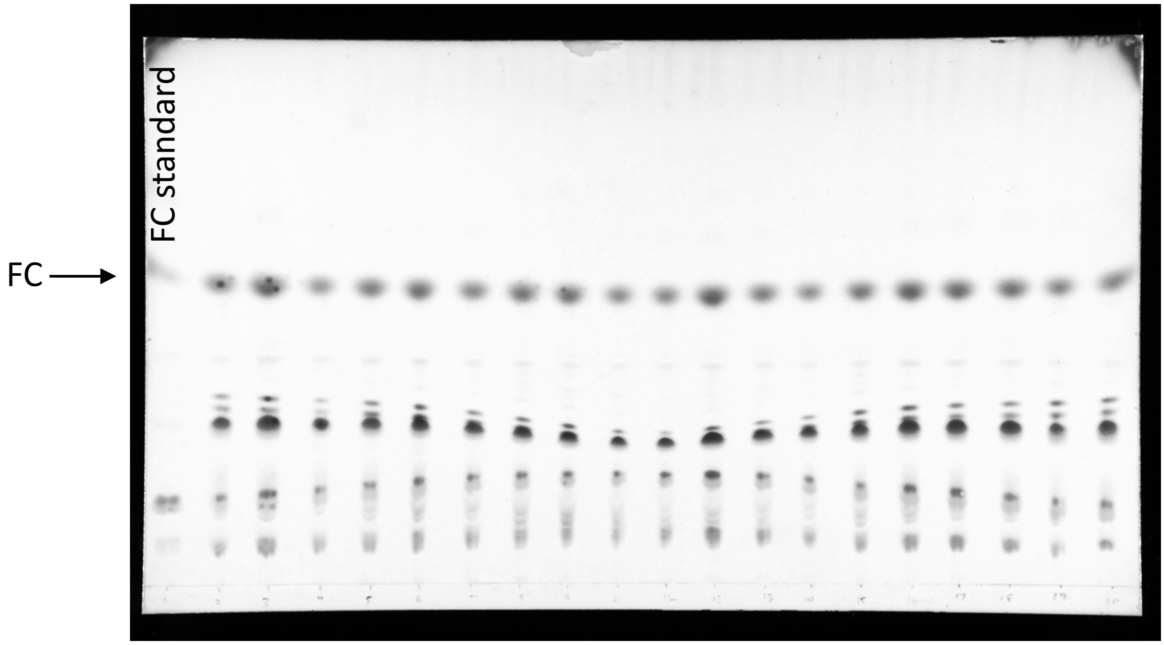

Supplementary Fig. 8 Thin layer chromatogram of female brain

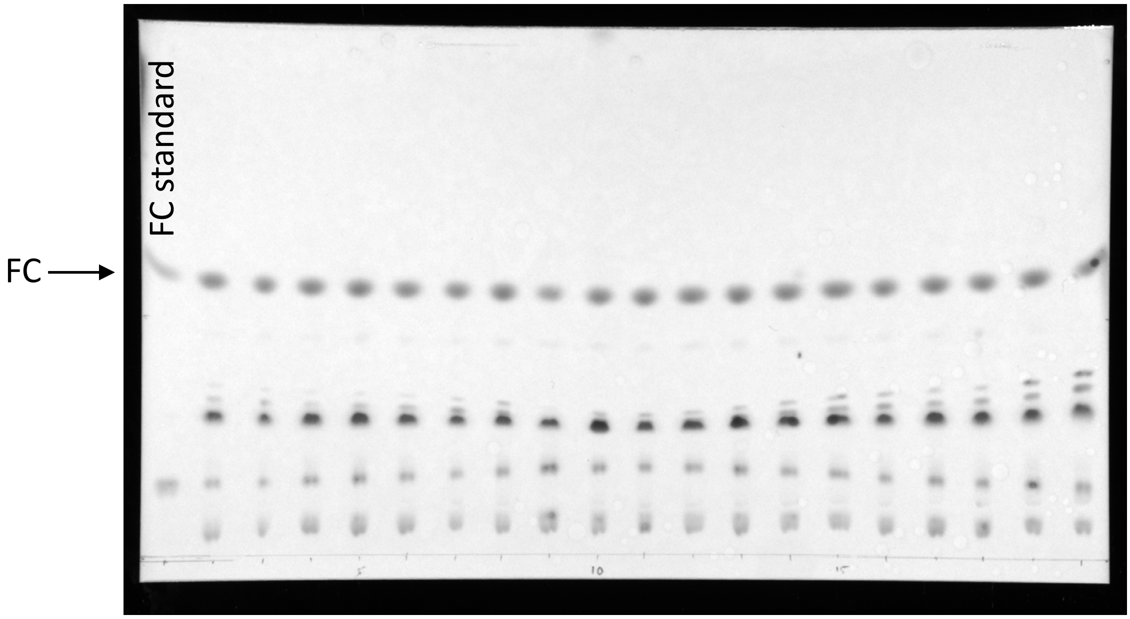

Supplement: fcac120_Supplementary_Data [file fcac120_supplementary_data.zip › Supplementary_figures.pdf]
